# Reaction mechanism of the farnesyl pyrophosphate *C*-methyltransferase towards the biosynthesis of pre-sodorifen pyrophosphate by *Serratia plymuthica* 4Rx13

**DOI:** 10.1038/s41598-021-82521-9

**Published:** 2021-02-04

**Authors:** Marie Chantal Lemfack, Wolfgang Brandt, Katja Krüger, Alexandra Gurowietz, Jacky Djifack, Jan-Philip Jung, Marius Hopf, Heiko Noack, Björn Junker, Stephan von Reuß, Birgit Piechulla

**Affiliations:** 1 Institute of Biological Sciences, University of Rostock, Albert-Einstein-Straße 3, 18059 Rostock, Germany; 2 Department of Bioorganic Chemistry, Leibniz-Institute of Plant Biochemistry, Weinberg 3, 06120 Halle, Germany; 3 Institute of Pharmacy/Biosynthesis of Active Substances, Hoher Weg 8, 06120 Halle (Saale), Germany; 4 Laboratory of Bioanalytical Chemistry, Institute of Chemistry, University of Neuchatel, Avenue de Bellevaux 51, 2000 Neuchâtel, Switzerland; 5 Department of Internal Medicine I, University Hospital RWTH Aachen, 52074 Aachen, Germany; 6 Institute of Biology, Martin-Luther-Universität Halle-Wittenberg, Weinberg 10, 06120 Halle (Saale), Germany; 7 PIMAN Consultants, 12 Rue Barthelemy Danjou, 92100 Boulogne-Billancourt, France; 8 Duale Hochschule Gera-Eisenach, Weg der Freundschaft 4, 07546 Gera, Germany

**Keywords:** Biochemistry, Chemical biology, Computational biology and bioinformatics

## Abstract

Classical terpenoid biosynthesis involves the cyclization of the linear prenyl pyrophosphate precursors geranyl-, farnesyl-, or geranylgeranyl pyrophosphate (GPP, FPP, GGPP) and their isomers, to produce a huge number of natural compounds. Recently, it was shown for the first time that the biosynthesis of the unique homo-sesquiterpene sodorifen by *Serratia plymuthica* 4Rx13 involves a methylated and cyclized intermediate as the substrate of the sodorifen synthase. To further support the proposed biosynthetic pathway, we now identified the cyclic prenyl pyrophosphate intermediate pre-sodorifen pyrophosphate (PSPP). Its absolute configuration (6*R*,7*S*,9*S*) was determined by comparison of calculated and experimental CD-spectra of its hydrolysis product and matches with those predicted by semi-empirical quantum calculations of the reaction mechanism. In silico modeling of the reaction mechanism of the FPP *C*-methyltransferase (FPPMT) revealed a S_N_2 mechanism for the methyl transfer followed by a cyclization cascade. The cyclization of FPP to PSPP is guided by a catalytic dyad of H191 and Y39 and involves an unprecedented cyclopropyl intermediate. W46, W306, F56, and L239 form the hydrophobic binding pocket and E42 and H45 complex a magnesium cation that interacts with the diphosphate moiety of FPP. Six additional amino acids turned out to be essential for product formation and the importance of these amino acids was subsequently confirmed by site-directed mutagenesis. Our results reveal the reaction mechanism involved in methyltransferase-catalyzed cyclization and demonstrate that this coupling of *C*-methylation and cyclization of FPP by the FPPMT represents an alternative route of terpene biosynthesis that could increase the terpenoid diversity and structural space.

## Introduction

Terpenoids are structurally diversified and represent the dominant class of natural products, with 80,000 members. They represent more than 30% of all compounds described in the Dictionary of Natural Products^1,2^. Mainly isolated from plants and fungi, they display a wide range of ecological and biological functions in all forms of life. Despite this large number and the impressive complexity and diversity of these compounds, they are commonly synthesized from only two isomers of C_5_ units, isopentenyl pyrophosphate (IPP) and dimethylallyl pyrophosphate (DMAPP). Both compounds derive either from the mevalonate (MVA) or 2-C-methyl-D-erythritol 4-phosphate (MEP) pathway^3,4^. The C_5_ building blocks are condensed by prenyl pyrophosphate synthases to form linear precursors of all terpenoids with different chain lengths including geranyl pyrophosphate (GPP, C_10_), farnesyl pyrophosphate (FPP, C_15_), geranylgeranyl pyrophosphate (GGPP, C_20_) and their respective isomers. These precursors serve as canonical substrates for terpene synthases. The reaction involves the formation of highly reactive carbocation intermediates that undergo diverse cyclization cascades to form a broad range of compounds comprising monoterpenes (C_10_), sesquiterpenes (C_15_) and diterpenes (C_20_)^2^. The resulting terpenes can undergo different additional decorating reactions, e.g. oxidation, methylation, esterification, or carbon elimination reactions, which further increase terpenoid diversity.

An aspect that has not yet been deeply investigated is the biological modification of the conventional building blocks and linear isoprenoid pyrophosphate precursors prior to the cyclization cascade of the terpene cyclases, which also leads to the expansion of the terpenoid repertoire. In 2007, Dickschat et al.^5^ described the first example of prenyl pyrophosphate (GPP) precursor modification in the biosynthesis of 2-methylisoborneol, a contaminant of drinking water. Later on, it was shown in more detail that during the biosynthesis of this volatile by *Streptomyces coelicolor*, a GPP *C*-methyltransferase (GPPMT) catalyzed the methylation of GPP to yield a non-canonical acyclic allylic pyrophosphate intermediate (2-methyl GPP), which is the substrate of the methylisoborneol synthase^6,7^. This finding led to the use of GPPMT in synthetic biology to increase terpenoid structures with potentially new flavors or biological activities^8,9^. Additionally, the C_5_ building blocks (IPP and DMAPP) can be mono- or dimethylated enabling the biosynthesis of C_11_, C_12_, C_16_, and C_17_ prenyl pyrophosphates^10^. Likewise, the biosynthetic pathway of the antitrypanosomal homoterpenoid longestin from *Streptomyces argenteolus* includes a methyltransferase that methylates homo-IPP to produce (3*Z*)-3-methyl IPP which, along with IPP, is selectively accepted as extender unit by a GGPP synthase homolog to yield the dimethylated intermediate of GGPP^11,12^. Finally, in Lepidoptera the C_6_ compounds homo-IPP and homo-DMAPP enable the formation of FPP analogs with 16, 17, and 18 carbons^13^.

In the last decade, it was shown that several *Serratia plymuthica* isolates produce the unique sesquiterpene sodorifen (1,2,4,5,6,7,8-heptamethyl-3-methylenebicyclo[3.2.1]oct-6-ene (C_16_H_26_)), a polymethylated bicyclic volatile compound, with the main producer being *S. plymuthica* 4Rx13^14–16^. The ecological role of sodorifen is so far unknown, but its production by the bacteria can be significantly up-regulated during interaction with other microorganisms^17,18^. Transcriptome and genome analysis of *S. plymuthica* 4Rx13 highlighted a cluster of four genes encoding for a terpene synthase, *C*-methyltransferase, DOXP synthase, and IPP isomerase^19^. Knockout studies of these genes by insertional mutagenesis showed that the terpene synthase (SODS), *C*-methyltransferase (FPPMT), and IPP isomerase are indispensable for the biosynthesis of sodorifen^19,20^. Further studies on the biosynthesis of sodorifen revealed that FPP is methylated by a *S*-adenosyl methionine (SAM)-dependent *C*-methyltransferase and that SODS does not accept FPP as a substrate in contrast to common sesquiterpene synthases^21^. Moreover, besides transferring a methyl group to carbon 10 of FPP, the FPPMT also exhibits a cyclase activity to produce the monocyclic compound pre-sodorifen pyrophosphate (PSPP). The FPPMT is the first known enzyme that catalyzes not only the methylation of FPP but also the cyclization of a prenyl pyrophosphate precursor in the biosynthesis of terpenoids. ^13^C isotope labeling experiments and NMR analysis revealed that the methylation of FPP leads to the formation of a five-membered ring, accompanied by several methyl or/and hydride shifts^21^.

To confirm the reaction mechanism of the FPPMT, we now identified this unusual cyclic prenyl pyrophosphate intermediate and elucidated the catalytic mechanism of the FPPMT involved in the formation of PSPP based on a high-quality protein model obtained from the Robetta protein folding Web Server with subsequent refinements and quantum chemical calculations. The model guided a multitude of site-directed mutagenesis studies that strongly support the correct structure of the protein model and the calculations of the catalytic mechanism. Furthermore, the absolute configuration of PSPP as predicted by the model and DFT calculations was experimentally confirmed by CD spectroscopy of the corresponding hydrolysis product pre-sodorifen.

## Results

### Identification of pre-sodorifen pyrophosphate (PSPP)

**Figure 1:**
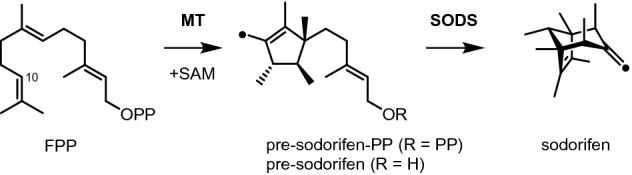
Biosynthesis of sodorifen from FPP catalyzed by a *C*-methyltransferase (MT) to afford cyclic PSPP as a non-canonical substrate for the terpene synthase (SODS). The black dot on pre-sodorifen and sodorifen indicate the methyl group transferred from SAM.

*Serratia plymuthica* 4Rx13 FPP *C*-methyltransferase (FPPMT) exhibits an unprecedented activity during the biosynthesis of the sesquiterpene sodorifen (Fig. 1). This enzyme catalyzes the methylation of FPP along with a cyclization reaction to produce a polymethylated compound with a five-membered ring named pre-sodorifen pyrophosphate (PSPP), which is a non-canonical substrate for the terpene synthase SODS^21^. Its molecular structure was derived by NMR analysis of its hydrolysis product (the alcohol pre-sodorifen) that accumulates in the terpene synthase knockout mutant. However, the presence of the corresponding pyrophosphate has so far only been deduced from coupled enzyme assays^21^.

**Figure 2:**
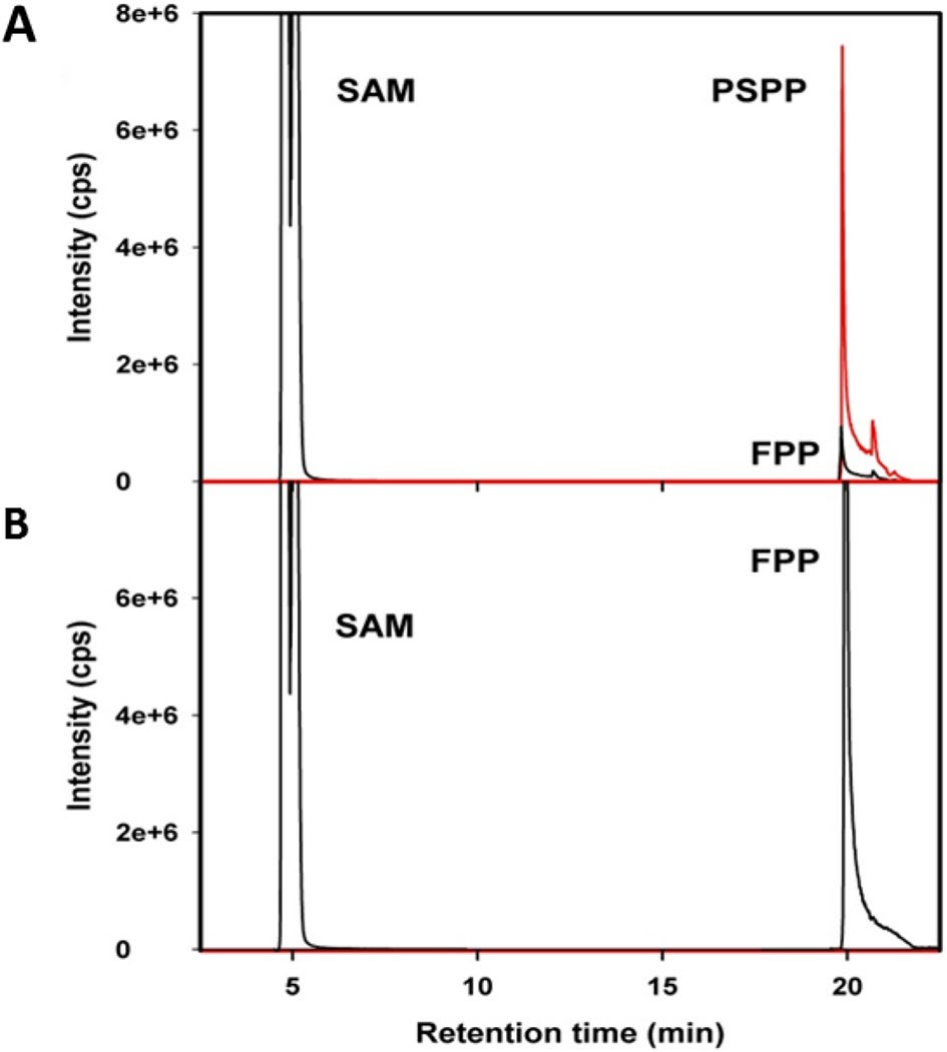
LC–MS separation of SAM, FPP, and putative PSPP in MRM-mode. Mass transitions are given in the method section. Enzyme assays including FPP and SAM were incubated, (**A**) with FPP *C*-methyltransferase enzyme (FPPMT), (**B**) without FPPMT. FPP and PSPP (red) elute at very similar retention times. Since no PSPP formation was observed in the control (**B**), the formation of PSPP (**A**) is enzyme-dependent. The split peak for SAM is due to detector overload.

Here we employed LC–MS to unambiguously establish the presence of this intriguing cyclic prenyl pyrophosphate intermediate which has a theoretical mass of *m/z* 395.1 Da. Direct infusion of the enzyme assay reaction mix into the mass spectrometer (scan mode) confirmed the presence of a mass peak with *m/z* 395.1 Da and fragmentation of the precursor ions furnished pyrophosphate and phosphate ions as fragments with major intensity (Fig. S1). Hence, for the LC–MS detection in multiple reaction monitoring (MRM) mode, we optimized substrate-dependent parameters for the fragmentation of PSPP to phosphate. LC–MS separation of the products of the enzyme assay mixture (FPP + SAM + FPPMT) revealed three peaks corresponding to S-adenosyl methionine (SAM), FPP, and PSPP. The retention time of PSPP was close to that of FPP (Fig. 2A). PSPP formation was FPPMT dependent since enzyme assays in the absence of FPPMT did not lead to any PSPP formation (Fig. 2B). This result corroborated with a significant reduction in the amount of FPP compared to the control enzyme assays (Fig. 2).

LC–MS analysis allowed us to compare the fragmentation patterns of putative PSPP and FPP (Fig. S2A,B). The pattern of FPP in the negative ionization mode is well known^22^ and was confirmed in our analysis. Apart from the most abundant mass fragments (phosphate and pyrophosphate), there was only one carbon fragment of FPP with sufficient intensity (C_15_H_23_O_6_P_2_ at *m/z* 363.1 Da) (Fig. S2B,C). In the fragmentation pattern of the putative PSPP, a carbon fragment at *m/z* 377.2 Da was observed. The additional methylene group (-CH_2_-) in the PSPP molecule resulted in a mass increase of about 14 Da compared to the corresponding carbon fragment of FPP.

### Protein structure model of the *Serratia plymuthica* FPP *C*-methyltransferase

**Figure 3:**
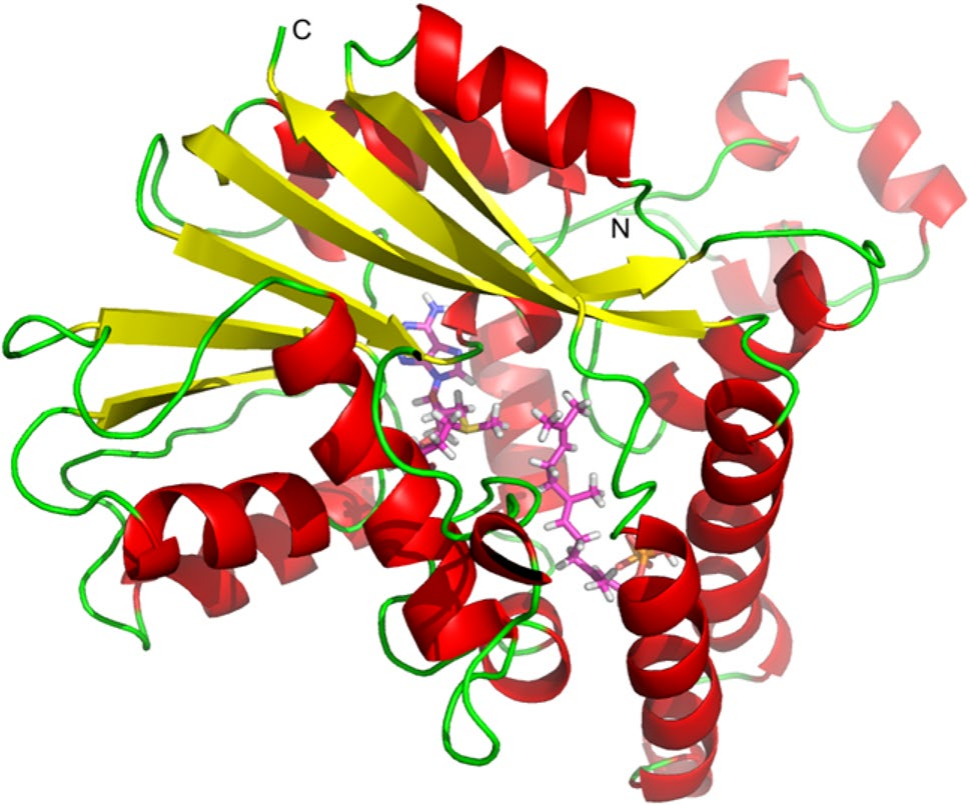
Tertiary structure model of the *Serratia plymuthica* FPP *C*-methyltransferase. SAM and FPP are docked into the center of the model (magenta carbon atoms).

To determine the catalytic reaction mechanism of the *S. plymuthica* FPPMT that converts FPP to PSPP, structural knowledge of the enzyme is a prerequisite. Since so far, its crystallization was not successful, we performed in silico homology modeling. Using the Robetta protein folding Web Server^23^, a model of the tertiary structure was obtained (Fig. 3). The co-factor SAM and the substrate FPP (magenta carbon atoms) were docked into the active pocket located at the center of the model. All criteria for a reasonable structure indicated by PROSA II and a PROCHECK analysis were fulfilled (Figs. S3, S4). The energy graphs were all in the negative area and a combined energy z-score of -10.15 by PROSA II indicated a native-like fold of the model.

**Figure 4:**
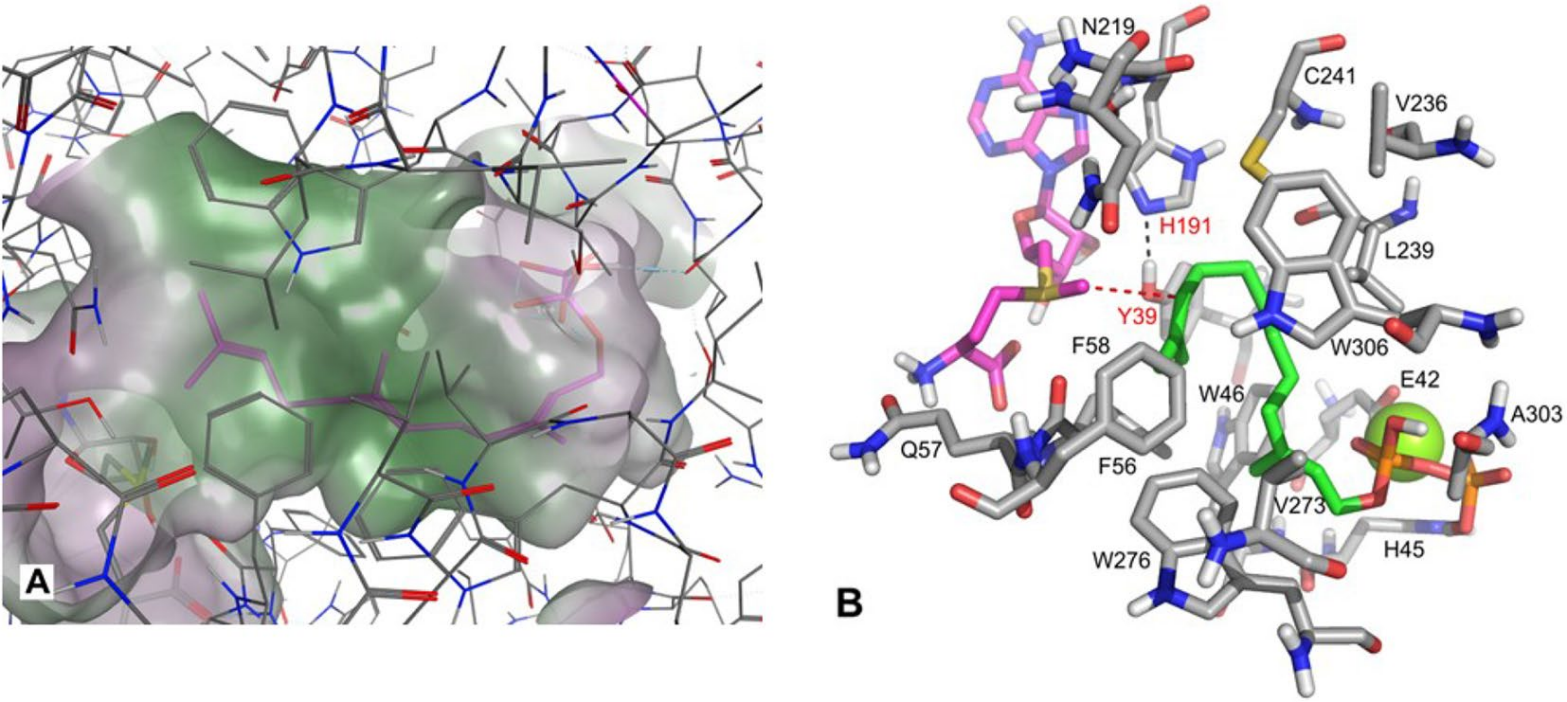
Binding site of FPP and stick model of the *Serratia plymuthica* FPP *C*-methyltransferase. (**A**) The binding site exhibits lipophilic potential (green area). (**B**) The starting structure of the active site was used for PM7 reaction coordinate calculations. All backbone atoms of the amino acid residues were fixed during all calculations. For better visualization, non-polar hydrogen atoms are not displayed. The substrate FPP is represented by green carbon atoms and SAM is shown in magenta. Green ball close to E42 represents Mg^2+^. Except for V236, V273, and F58, all labeled amino acid residues were experimentally mutated to alanine (Tables 1, S3), which resulted in inactivation or reduction of enzymatic activity.

The best scored docking pose that fulfills a close distance (3.1 Å) of the SAM methyl carbon atom to the carbon atom at position 10 of FPP was used as starting structure for all the semi-empirical quantum mechanical PM7 calculations. The entire structure can be downloaded from the IPBs home page^24^. The binding site of FPP is characterized by a very narrow hydrophobic environment (Fig. 4A) and is formed by W46, F56, F58, L239, V273, and W306 (Fig. 4B). The diphosphate moiety of FPP is recognized by a magnesium ion ligated by E42 and forms a hydrogen bond with the protonated H45. A catalytic dyad is formed by Y39 and H191 and was identified to be of high importance for the catalytic mechanism.

### Proposed catalytic mechanism of the formation of pre-sodorifen pyrophosphate

**Figure 5:**
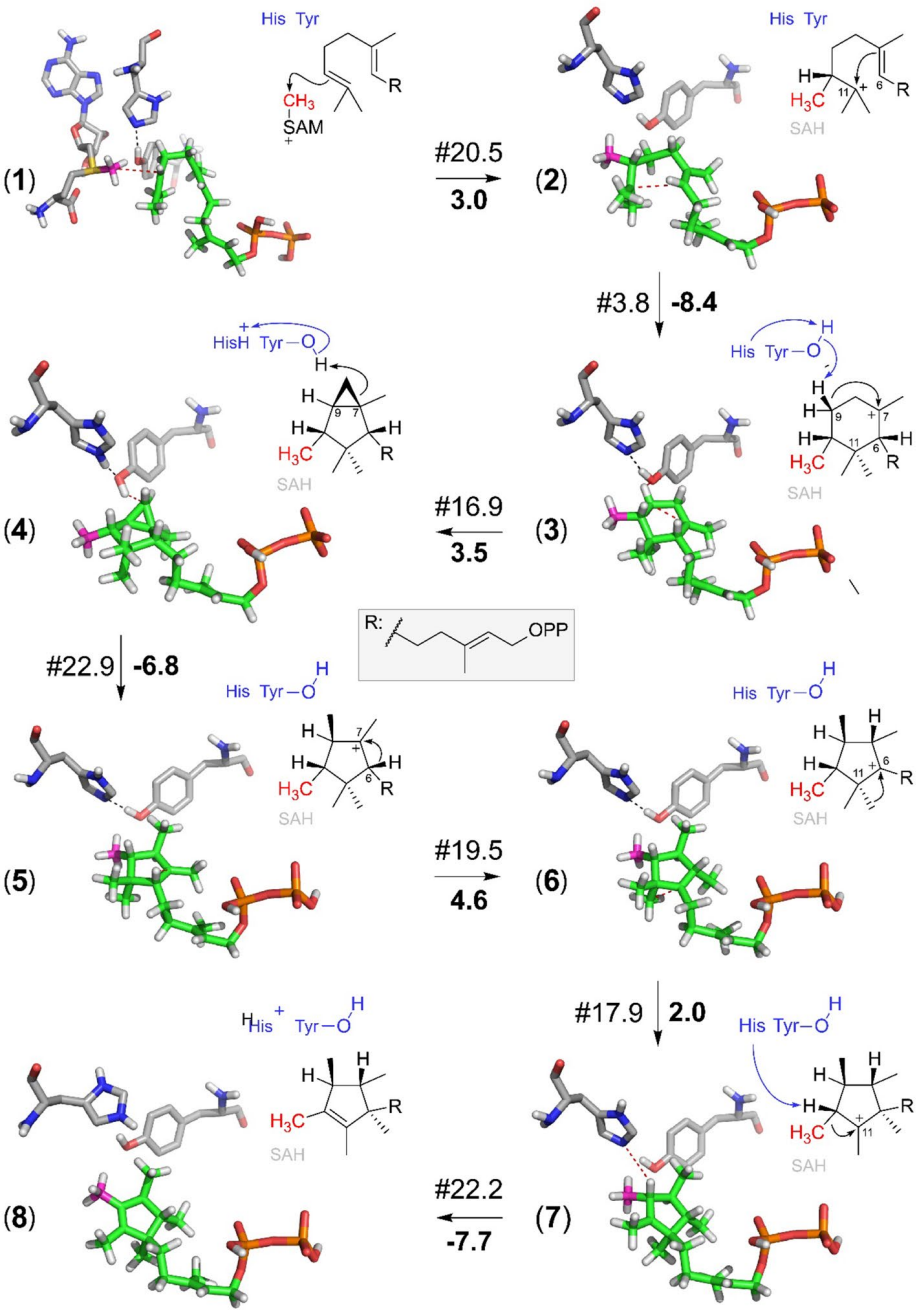
Intermediate steps of the catalytic mechanism for the formation of PSPP by the *Serratia plymuthica* FPP *C*-methyltransferase. Intermediate steps of the catalytic mechanism for the formation of PSPP by the *Serratia plymuthica* FPP C-methyltransferase. The PM7 calculations include all atoms of the active site model shown in Fig. 4B. For better visualization only the essential parts are displayed, thus non-polar hydrogen atoms that are not required for the understanding of the mechanism and the formed SAH in reaction steps (2–8) are not displayed in the 3D representations. Below each reaction arrow, the reaction enthalpy is given (in kcal/mol). Above each reaction arrow, the required activation energy (kcal/mol) is indicated by “#”. The reaction starts with the methyl-transfer from SAM (magenta carbon atom) (**1**) to the C10 carbon atom of FPP (green carbon atoms). The carbon atom colored in gray attached to FPP is in all 3D figures (steps **2–8**) the one that has been transferred from SAM. Red dotted lines indicate the reaction coordinates. Histidine (H191) and tyrosine (Y39) are the amino acids that form the essential dyad.

Semi-empirical quantum mechanical PM7 calculations were performed based on the starting model of the active site (Fig. 4B). The calculation rate of this method is rather high, particularly when, as in this case, a molecular system with 424 atoms must be calculated. On the other hand, the average error in heat formation for PM7 has been given as less than 4 kcal/mol^25^. Thus, the method is appropriate for studying a multitude of alternative reaction pathways to predict the most likely one. The entire catalytic mechanism is summarized in Fig. 5.

Based on in vitro enzyme assays and incorporation experiments with ^13^C labeled precursors a reaction mechanism has previously been proposed for the formation of PSPP from FPP that commences with the transfer of the methyl group of SAM to the C10-carbon atom of FPP^21^. The energy of the optimized starting structure (**1** Fig. 5) as relative reference energy was set at 0.0 kcal/mol, an activation barrier (the inversion of the leaving methyl cation) of 20.5 kcal/mol has to be passed. This rather high barrier of the initial step is not unusual for methyltransferases^26^. The energy of the C10-methylated farnesyl cation complex (**2**) is 3.0 kcal/mol higher than the energy of the starting complex. However, in the next reaction step the dimethyl cation (C11) is in close distance (3.2 Å) to the C6-carbon atom of the double bond thus a six-membered ring (**3**) can be easily formed and leads to an energy gain with respect to the starting complex of -8.4 kcal/mol accompanied with a very low barrier of 3.8 kcal/mol. For alternative reactions, deprotonation from either terminal methyl group seems not to be possible as this could potentially only happen from the methyl group next to the cationic center. The formation of PSPP required a conversion of the six-membered ring into a five-membered ring, with several hydrid- and methyl-shifts. The hypothesis whether the formation of an intermediate cyclopropyl with subsequent opening can lead to an energetically favored formation of the five-membered ring was tested by calculation. The distance between the C9-carbon atom to the C7-carbocation was stepwise shortened. In a distance of 1.9 Å (representing the transition state of 16.9 kcal/mol) between both atoms one of the protons at C9 jumps without barrier to the phenolic hydroxyl group of Y39. Y39 was identified as part of a catalytic dyad with H191. H191 serves as proton acceptor from Y39. Thus without barrier the intermediate cyclopropyl moiety is formed instantaneously (**3** to **4**). Since a cyclopropyl ring has high ring constrain the reaction is slightly disfavored by 3.5 kcal/mol with an activation barrier of 16.9 kcal/mol, thus subsequently it can be opened by reprotonation from Y39 to C8 (**4**) with an energy gain of -6.8 kcal/mol and a barrier of 22.9 kcal/mol to form structure **5**.

Subsequently, a hydride transfer from C6 to C7 (**5** to **6**, energy effort 4.6 kcal/mol, activation barrier 19.5 kcal/mol) followed by a methylanion transfer of C15 from C11 to C6 (**6** to **7**, energy effort 2.0 kcal/mol, activation barrier 17.9 kcal/mol) led to the correct relative stereochemistry of the intermediate **7** (*cis* methyl groups at C6 and C7 and *trans*-configurations to the migrated hydrid at C7). The theoretically alternative transfer of the equatorial C12 instead of C15–C6 is disfavored by 4 kcal/mol and requires the passage of an energy barrier of 24 kcal/mol. Since a proton of C10 (where the former methyl group of SAM was attached) was in close proximity (1.86 Å) to the N-atom of H191, no other alternative pathway seemed to be favored except the obvious proton abstraction by H191 accompanied with an energy gain of − 7.7 kcal/mol to form PSPP. Altogether, the entire catalytic mechanism was thermodynamically favored by − 9.5 kcal/mol. All the positions of the carbon atoms are in agreement with the ones derived and experimentally determined including the *cis*-C14–C15 configuration. In summary, among the previously suggested reaction mechanisms^21^, the shown pathway in Fig. 5 is strongly favored and supported by detailed quantum mechanical calculations and the identification of the catalytic dyad Y39-H191. The graphical representation of the complete energy profile is shown in Fig. S5.

Several catalytic mechanisms and pathways as well as terpenoid formation have already been studied by quantum mechanical methods^27,28^ and the application of the density functional theory like DFT (B3LYP) was demonstrated to be appropriate for carbocation reactions involving terpenes. Therefore, to evaluate and support the results of the semi-empirical PM7 calculations more advanced DFT calculations have been performed. Single point energies for all the intermediates and corresponding transition state structures (Fig. 5) were calculated using a smaller model of the active site (Fig. S6). The results are summarized in Table S2. The relative energies showed some slight differences in comparison to the PM7 calculations in between each reaction step and in the transition state energies. These differences may result from the usage of a truncated model of the active site. However, the energy gain of − 19.4 kcal/mol obtained by the DFT calculations for the entire reaction supports the suggested mechanism.

### Determination of the stereochemistry of pre-sodorifen pyrophosphate by circular dichroism spectroscopy

The enzymatic cyclization of the achiral FPP to the chiral PSPP by the FPPMT involves the induction of chirality, which facilitates the comparison of the predicted and experimental data. Based on the quantum mechanical calculations of the catalytic reaction pathway the absolute stereochemistry of PSPP was predicted to be 6*R*,7*S*,9*S*, which prompted us to determine its absolute stereochemistry using circular dichroism spectroscopy.

**Figure 6:**
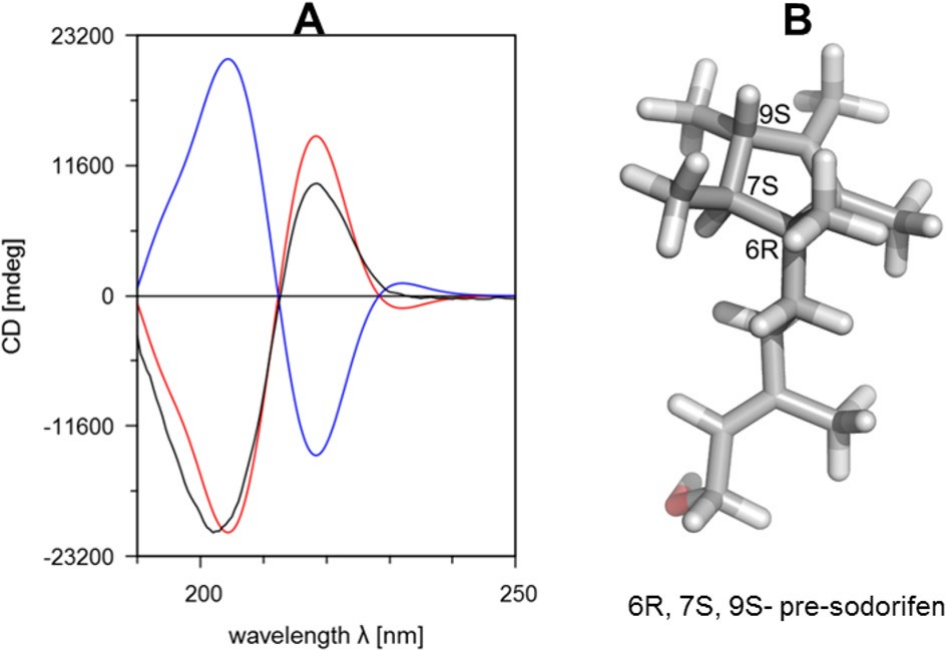
Determination of the stereochemistry of pre-sodorifen from the *Serratia plymuthica* sodorifen synthase (SODS) knockout mutant. (**A**) The CD spectrum (black line) coincides with the calculated CD-spectrum of 6R, 7S, 9S-pre-sodorifen (red line, similarity factor = 0.9972) while its enantiomer (blue line) does not. (**B**) Observed stereochemistry of pre-sodorifen.

Since PSPP has not yet been isolated, CD spectroscopy was performed with the corresponding alcohol pre-sodorifen. Its production is strongly upregulated in the *S. plymuthica* terpene synthase (SODS) knockout mutant^20^. The CD-spectrum of pre-sodorifen in 6*R*,7*S*,9*S* configuration was calculated with DFT using the B3LYP functional and subsequently compared with the experimentally obtained one. The CD spectrum of the natural product matched well with those calculated for (6*R*,7*S*,9*S*)-pre-sodorifen (but not with those of the 6*S*,7*R*,9*R* enantiomer) (Fig. 6). This is in agreement with the absolute stereochemistry predicted by in silico modeling of the reaction mechanism of the FPP *C*-methyltransferase.

### Experimental validation of the structure model by site-directed mutagenesis

The in silico homology modeling identified amino acids that form the active pocket and the modeling studies of the catalytic reaction mechanism suggested their putative functions. The importance of these amino acids was subsequently confirmed by site-directed mutagenesis. Almost all amino acid residues of the active site (Fig. 4B) were experimentally replaced by alanine by a single nucleotide mutation. The resulting mutant enzymes were tested in double (FPPMT + SODS, product: sodorifen) or coupled enzyme assays (FPPMT + alkaline phosphatase, product: pre-sodorifen). In total twenty-six amino acids were mutated to alanine (Tables 1, S3). Among these mutations, 12 were located outside of the active site and did not show any change in enzyme activity (Figs. S7, S8, S9). All 14 mutations located within the active center led to a strong reduction or loss of enzyme activity as almost no products (sodorifen or pre-sodorifen) could be detected (Table 1, Figs. S10, S11). Mutations of the amino acids W46, F56, and W306, proposed to stabilize the carbocation and form a hydrophobic environment in the binding site of FPP, resulted in completely inactive enzymes. Also, the enzyme assays performed with the mutated amino acids of the catalytic dyad, Y39 and H191, did not lead to any product. Altogether, the GC–MS analysis of the assay products obtained from all these 5 mutant enzymes showed no detectable sodorifen or pre-sodorifen (Figs. S10, S11). H45 and E42 were proposed to be involved in magnesium cation binding which interacts with the diphosphate moiety of FPP. Their mutation to alanine led to an inactive enzyme (E42A) or drastically reduced activity (H45A). In the case of E42A there were no detectable products upon GC–MS analysis while H45A results in ca. 25% of sodorifen and pre-sodorifen compared with the product profile of the wild type enzyme (Table 1). Moreover, mutations of the amino acids L239 and C241, located in the hydrophobic active pocket near the farnesyl moiety (particularly L239), also resulted in a strong reduction of enzymatic activity. Only ca. 20% of sodorifen and pre-sodorifen was produced by these mutant enzymes. Interestingly, the mutant enzymes L239A and C241A led to the production of six different but new compounds (#1, 2#, and 3# in the double assays and #4, 5#, and 6# in the coupled assays) (Figs. S12, S13). However, the molecular structures of these potential derailment products remain so far unidentified.

**Table 1:** GC–MS analysis of the enzyme assay products.

Amino acid	Putative function	Mutant	Sodorifen	pre-sodorifen production
**Amino acids of the active site**				
Y39	Catalytic base, catalytic dyad	Y39A	No	No
E42	Mg^2+^ coordination	E42A	No	No
H45	Pyrophosphate stability	H45A	25% ± 18%	25% ± 11%
W46	Carbocation stabilization, hydrophobic pocket	W46A	No	No
F56	Carbocation stabilization, hydrophobic pocket	F56A	No	No
Y61	Correct position of SAM in the active site	Y61A	No	No
H191	Catalytic base, catalytic dyad	H191A	No	No
N219	Closes active site and helps correct positioning of FPP	N219A	No	No
D237	Enzyme stability	D237A	No	No
L239	Hydrophobic pocket, enzyme stability	L239A	20% ± 10%	23% ± 14%
C241	Enzyme stability	C241A	20% ± 8%	22% ± 15%
D272	Enzyme stability	D272A	No	No
E297	Stabilizes tertiary structure for correct recognition of pyrophosphate	E297A	25% ± 17%	27% ± 7
W306	Carbocation stabilization, hydrophobic pocket	W306A	No	No
Wild Type			100% ± 8%	100% ± 3%

Double (for sodorifen production) or coupled enzyme assays (for pre-sodorifen) were performed with wildtype and mutant FPP *C*-methyltransferase of *Serratia plymuthica.*

## Discussion

Pre-sodorifen pyrophosphate (PSPP) represents an unprecedented cyclic prenyl pyrophosphate proposed to serve as intermediate in the biosynthesis of the sesquiterpene sodorifen^21^. Using LC–MS analysis it was now possible to detect and analyze this biosynthetic intermediate for the first time. After fragmentation in the MS, the major carbon fragment of PSPP exhibited a mass increase of 14.1 Da compared to FPP, indicating the presence of an additional methylene group (-CH_2_). Ion pair chromatography showed that the retention time of the PSPP is very close to that of FPP. This is due to the identical phosphate content and similar lipophilicity, as the latter are critical separation parameters of the ion pair chromatography. Moreover, the amount of FPP was significantly reduced (Figs. 2, Fig. S3) in the assay mixture incubated with FPP *C*-methyltransferase (FPPMT). This strongly indicated that PSPP formation is due to the conversion of FPP by the FPPMT enzyme. In conclusion, the identification of PSPP is in good agreement with the proposed reaction pathway involving both methylation and cyclization of FPP catalyzed by the SAM-dependent FPPMT^21^.

In silico modeling of the catalytic mechanism of FPPMT showed that the biosynthesis of PSPP is initiated by the formation of a carbocation due to the S_N_2-like *C*-methylation of FPP. Carbocation formation is the typical initiation reaction for terpenoid biosynthesis in plants and microorganisms. Its formation is commonly initiated by conserved aspartate-rich motifs, present in the active site of the terpene cyclases, that bind cation and trigger the departure of the diphosphate moiety of the FPP to form a carbocation that will undergo the cyclization cascade^2^. The aspartate-rich motifs were not identified in FPPMT and although E42 and H45 are suggested to bind a magnesium cation that interacts with the diphosphate moiety of FPP, the carbocation is generated by methylation instead of elimination of the diphosphate moiety. Moreover, the subsequent cyclization cascade of this carbocation towards the formation of the five member-ring of PSPP is guided by the His-Tyr (H191 and Y39) catalytic dyad localized near the reactive methyl group of SAM. A His-Tyr dyad closely located to the reactive methyl group of SAM was also reported for the phosphoethanolamine N-methyltransferase from *Plasmodium falciparum* and is required for the methylation of phosphoethanolamine to phosphocholine. In the latter case, no cyclization reaction occurred and the dyad formes a catalytic lid that locks ligand in the active site while D128 deprotonated the substrate via a bridging water molecule followed by a typical SN_2_-type methyl transfer from SAM to phosphoethanolamine^29,30^. The only known methyltransferase with cyclization activity is the TleD methyltransferase of *Streptomyces blastmyceticus*^31^. The X-ray crystal structure of TleD methyltransferase, which induces a cyclization reaction after methylation in the biosynthesis of the antibiotic teleocidin B, requires a hydrogen bond between Y21 and H157. Both amino acids were shown not to be involved in the cyclization cascade but play the most important role in maintaining the local protein fold which is important for the TleD enzyme activity^32^. Therefore, the catalytic dyad of FPPMT is highly specific because the acceptance of a proton by the dyad does not terminate the reaction but instead, the proton is used to (re)-protonate the cyclopropyl moiety, which initiates ring opening followed by another rearrangement cascade like in terpenoid cyclization reactions (Fig. 5). Histidine and tyrosine perform general acid–base mediated catalysis in the cyclization mechanism of terpene cyclases. In the active site of the tobacco 5-*epi*-aristolochene synthase, for example, Y520 forms with two aspartate residues a catalytic triad, involved in the cyclization reaction of germacrene A during the biosynthesis of the sesquiterpene 5-*epi*-aristolochene^33^. Likewise, the side chain of H232, an essential and conserved residue among the oxidosqualene cyclases, is suggested as the only base that accepts a proton in the active site of lanosterol synthase to terminate the cyclization cascade during the biosynthesis of the triterpene lanosterol^34^. Besides this catalytic dyad (H191 and Y39), the amino acids W46, W306, F56, and L239 of the FPPMT create a hydrophobic environment for the substrate’s lipophilic tail (farnesyl moiety) and the aromatic side chains of F56, W306 and W46 may stabilize the carbocation intermediate during the rearrangement reactions. This is also consistent with the binding site of prenyl pyrophosphate substrates of terpene synthases where the lipophilic tail of the substrate is buried in a hydrophobic pocket. During the cyclization cascade of the terpene cyclase, the carbocation intermediates can be stabilized by the π electrons of the aromatic ring of amino acids like phenylalanine, tyrosine, and tryptophan through cation − π interactions^2^. Such a hydrophobic active site is also found in prenyl pyrophosphate methyltransferases such as IPPMT of *Streptomyces monomycini* and GPPMT of *Streptomyces coelicolor*^10,35^. The X-ray crystal structure of GPPMT showed that the binding site of GPP is defined by the side chains of several aromatic and other hydrophobic amino acids that favor the binding of prenyl pyrophosphate substrate^35^. Especially, the side chain of F222 was shown to stabilize the carbocation by cation-π interactions and by electrostatic interactions with the side chain of E173, while each phosphate group of the substrate (GPP) was coordinated to a single Mg^2+^ ion complexed by the side chain of N37. Nevertheless, the amino acids Y51 and H221 found in the active pocket of GPPMT were shown to not be involved in any acid–base catalysis^35^. Furthermore, GPPMT showed a very low sequence identity (16.9%) with FPPMT and also turned out to be an unsuitable template in the FPPMT homology modeling studies^36^. In summary, it seems likely that the amino acid Y39 and H191 which form the catalytic dyad of the *Serratia plymuthica* FPPMT are conserved in several methyltransferases (Fig. S14) and could be very important for the methylation reaction. However, the function of the catalytic dyad (important for the cyclization reaction during the formation of PSPP) seems not to be conserved and is evolved in FPPMT.

Furthermore, site-directed mutagenesis exchange of the amino acids Y61, N219, D237, D272, L239, C241, and E297 to alanine led also to a strong reduction or loss of the FPPMT enzymatic activity. These amino acid residues are all closely located in the active site of the enzyme, most likely being important for the catalysis, e.g. Y61 could stabilize the correct positioning of SAM in the active site. Since the mentioned amino acids have the potential to form H bonds it can be speculated that they play a role in protein stability and help to form a high-fidelity active site for the PSPP biosynthetic mechanism. The mutants L239A and C241A were particularly interesting since new compounds were produced, the structures of which were so far not elucidated. Their mass spectra exhibited some similarities (Figs. S15, S16) with those of sodorifen, pre-sodorifen, or related compounds observed in *S. plymuthica* VOC profile, suggesting that these compounds might be intermediates of the cyclization cascade during the biosynthesis of PSPP. The elucidation of their structures will provide additional insights into the biosynthesis of PSPP. It will be also important to perform X-ray crystal structure and CD spectra analyses of the products of the FPPMT mutants compared to those of the wildtype (ongoing work), to support the suggested function of the targeted amino acids. In summary, the cyclization reaction catalyzed by FPPMT is similar to that of terpene cyclases and is particularly intriguing since no analogous reaction has been reported that includes cyclic prenyl pyrophosphate substrates for terpene synthases. In this context and given the fact that the SODS does not accept FPP as a substrate, it is an interesting but presently unsolved question why both FPPMT and SODS co-evolved to catalyze such a complex and unique reaction to produce sodorifen, as the ecological and biological function of sodorifen has also not yet been solved.

It is known that SAM-dependent methyltransferases appear ubiquitous in all branches of life and are involved in a multitude of biological reactions^37–40^. Five structurally distinct classes have been described and the largest majority of known methyltransferases belong to class I^41,42^. They have a characteristic structure called Rossmann‐like superfold, consisting of alternating β-strands and α-helices, which form an αβα sandwich structure^37,38^. The tertiary structure model of the FPPMT obtained from the Robetta protein folding Web Server shows similar structural features as it consists of the αβα fold. These results strongly indicated that FPPMT belongs to the class I methyltransferases. Early studies of the structural features of class I methyltransferases showed that they are a good example of convergent evolution in enzymes as the SAM core fold is highly conserved among them despite the low overall methyltransferase amino acid sequence similarity^37,41–43^. Alignment of FPPMT with 14 other microbial class I methyltransferases revealed also low sequence similarity^36^. Nevertheless, the conserved GxGxG SAM-binding motif of class I methyltransferases^37^ is formed by the amino acids G114, G116, and G118 of the FPPMT (Fig. S14). While the core SAM binding motif is highly conserved, the binding site of class I methyltransferase substrates varies considerably according to the nature of the respective molecule. It also has to be kept in mind that methyltransferases can transfer a methyl group on the carbon, oxygen, nitrogen, or sulfur atom of a broad range of substrates varying from macromolecule like lipids, proteins, nucleic acids, hormones to small molecules like catecholamine^37–40^. However, FPPMT is the first methyltransferase that accepts FPP as a substrate, consequently, its binding pocket does not share (conserved) similarities with other substrate binding sites. Recent data clearly provide evidence for specialized bacterial *C*-methyltransferases that accepted prenyl pyrophosphates like IPP, DMAPP, and GPP as substrates. They catalyzed the biosynthesis of a variety of methylated non-canonical substrates for the subsequently acting terpene synthases^5–7,10,12,13^. While GPPMT catalyzed the methylation of GPP followed by subsequent deprotonation of the carbocation to produce 2-methyl-GPP, FPPMT catalyzed a methylation at carbon 10 of FPP followed by a cyclization to synthesize PSPP as the substrate for the SODS. Although FPP and GPP are terpenoid building blocks and undergo *C*-methylation, the catalytic mechanisms of both enzymes are completely different. It could be speculated that prenyl pyrophosphate methyltransferases adapted to the lipophilic compounds and with the size of these substrates (long hydrocarbon chain and more double bonds), they evolved to catalyze intramolecular cyclization reaction as well, similar to the cyclization reactions in sesquiterpene biosynthesis.

Altogether, the findings of this new type of *C*-methyltransferases, involved in terpenoid biosynthesis, strongly indicate that substrates for terpenoid biosynthesis are more diverse than previously expected. Such substrate variations greatly increase the structural diversity of terpenoids as shown for some GPP methyltransferases when they were used in synthetic biology to increase terpenoid structures with potentially new/interesting properties^8,9,41^. It is also interesting to note that the multiple finding of prenyl pyrophosphate methyltransferases highlights a new dimension of substrate promiscuity of the corresponding specialized terpene cyclases in bacteria^8,9,44^. Therefore, bacteria may be able to diversify and increase the terpenoid structural space by using non-canonical substrates. It is yet not known if this is an evolutionary old or recent adaptation for terpenoid metabolism. So far, methylated or cyclized prenyl pyrophosphate substrates of terpene synthases are only found in the prokaryotic domain as these specialized methyltransferases have yet not been discovered in plants and animals.

## Methods

A search in the protein database (PDB) for homologous proteins to the FPPMT revealed only three proteins ((pdb-code: 5KOK: the pavine N-methyltransferase^45^; 5DOO: a protein-lysine methyltransferase from *Rickettsia*^46^, and 3MGG: a methyltransferase from *Methanosarcina mazei*^47^), however, with very low sequence identity (5KOK 16.6%, 5DOO 14.8%, 3MGG 12.7%). This very low sequence identity was not sufficient for standard protein homology modeling. Therefore, the sequence was submitted to the Robetta Web Server^23^ for ab initio modeling or/and threading. Five models were returned. Their quality for putative native fold was checked with PROSA II and stereochemical quality with PROCHECK. The one with the lowest z-score value from the PROSA II analysis (Fig. S3) was used for further studies. The structure was superimposed on the X-ray structure of 5KOK which contains S-adenosyl-L-homocysteine (SAH) as a cofactor in its structure. The tertiary structure of the Robetta-model coincides very well in the central part of the enzyme (Fig. S17), which supports highly the principal correctness of the model. Therefore, SAH could be appropriately merged into the model without any difficulties. SAH was manually modified to SAM by replacing the hydrogen atom with a methyl group using MOE (molecular operating environment 2016.08, chemical computing group Inc., Montreal, QC, Canada) and subsequently submitted to 20 steps of simulated annealing md-refinement using YASARA^48,49,50^. The quality of the final optimized structure was again checked with PROSA II^51,52^ and PROCHECK^53^ (Figs. S3 and S4). 100 docking runs of FPP were performed with GOLD^54,55^. For the definition of the docking site, the methyl carbon atom of SAM was defined as origin with a radius of 15 Å. The docking results were evaluated with ChemPLP score^56,57^. The side chains of W46 and F56 were considered as flexible according to the rotamer library of GOLD. The dockings were inspected for appropriate orientation of the terminal prenyl moiety to the SAM methyl carbon atom, i.e. the distance of this carbon atom to the C10-carbon atom was measured. Only two very similar docking arrangements fulfilled the cut-off criterion of a distance smaller than 4 Å. Since in both docking positions the diphosphate moiety was located close to the side chains of E42 and a protonated H45, a magnesium ion was manually included between the side chain of E42 and the diphosphate moiety. Both structures were finally optimized with Amber 14:EHT force field^58,59^ in MOE keeping the backbone atoms of the protein fixed. The lipophilic potential (Fig. 6a) was also calculated with MOE.

Starting with both docking arrangements a multitude of semi-empirical quantum mechanical reaction coordinate calculations using PM7^60^ included in MOPAC2016^25^ were performed. For this purpose, the active site was cut from the protein structure model (see Fig. 4B). All backbone atoms were fixed during all quantum mechanical calculations to avoid distortions from the tertiary structure of the protein.

For optimization, the Broyden-Fletcher-Goldfarb-Shanno (BFGS) algorithm was used^61–64^. Performed scan and grid calculations were done with a step size of ± 0.2 Å. For each reaction coordinate (scan) or pair of reaction coordinates (grid), the final heat of formation (ΔH_f_) of the system was calculated directly resulting in an energy profile (scan) or an energy hyperplane (grid) from which the corresponding energy pathway was extracted.

Grids and scans were analyzed in MOE 2016.08 using a set of svl-scripts implemented by Richard Bartelt^65^. All images of the 3D-structures were rendered with PyMOL^66^.

All the intermediates as well as the corresponding transition state structures (Fig. 5) were directly taken from the PM7 optimized structures for single point energy calculations by more advanced energy optimizations using the density functional theory (DFT) implemented in the ab initio ORCA 4.0.0.2 program package^67^. The optimizations were done with the B3LYP functional with the def2-TZVP(-f) basis set and TightSCF optimization^68–71^. To save computational time, the diphosphate binding site was removed and a truncated model of the active site was used (F86, Y39, H191, L239, see Fig. S6). Therefore, instead of FPP, farnesyl alcohol was used to form pre-sodorifen in the last step of the reaction. All the backbone atoms and the hydroxyl group of the farnesyl alcohol were fixed during the optimization. Furthermore, SAM was fixed except of the three carbon atoms bound to the sulfur atom. The results are summarized in Table S3.

### Measurement of the CD spectrum

Pre-sodorifen was obtained from the *S. plymuthica* terpene synthase mutant as previously described^21^. The CD measurement in n-hexane was performed using a Jasco J-715 spectrometer (JASCO, Deutschland GmbH).

### Calculation of spectrum

The structure of pre-sodorifen that resulted as the product of the reaction mechanism calculation was optimized by applying the density functional theory (DFT) using the B3LYP functional with the SV (P) basis set^68–71^ implemented in the ab initio ORCA 3.0.3 program package^72^. The influence of the experimentally used n-hexane solvent was included in the DFT calculations using the COSMO model^73^. The quantum chemical simulations of the CD spectra were also carried out using ORCA. Therefore, the first 30 excited triplet states of the structure were calculated by applying the long-range corrected hybrid function TD B3LYP/G with SV (P). The CD-spectra of the corresponding enantiomer was obtained by mirroring from the calculated spectrum. The CD curves were visualized and compared with the experimental spectra with the help of the software SpecDis 1.64^74^.

### Site-directed mutagenesis

The *Serratia plymuthica* 4Rx13 FPP methyltransferase (SOD_c20760) and terpene synthase (SOD_c20750) genes were cloned into the Champion pET151/D-TOPO vector (Thermo Scientific, St. Leon-Roth, Germany)^21^. Nucleotide changes on the genes were generated using a modified Quick Change Site-Directed Mutagenesis Kit (Agilent, Böblingen, Germany) according to the manufacturer’s recommendation. Modifications of the protocol were: the pfu Ultra HF DNA polymerase and DpnI of this kit were replaced by a Phusion DNA polymerase (2 U/µL) and DpnI (10 U/µL) from Thermo Scientific (St. Leon-Roth, Germany) respectively. PCR parameters: 2 min initial denaturation at 98 °C was followed by 16 cycles of denaturation at 98 °C for 30 s, annealing at 65 °C for 60 s, and elongation at 72 °C for 14 min. Reactions were finished by a final elongation of 72 °C for 10 min. Each amino acid of interest was changed to alanine and the primers used are shown in the supplement (Table S1). The digestion of the methylated parental DNA template was performed by adding 0.5 µL of DpnI restriction enzyme to the PCR reaction tubes. The digestion was carried out for 1 h at 37 °C. Eighty ng of the mutated plasmid were used for the transformation of *E. coli* XL-blue cells. Stocks were stored at − 70 °C. Plasmids were re-isolated from single *E. coli* XL-blue clones using the NucleoSpin Plasmid Easy Pure Kit (Macherey–Nagel, Düren, Germany), and mutated sequences were confirmed by Sanger sequencing (Eurofins GATC Biotech, Konstanz, Germany).

### Heterologous expression and purification of proteins

The proteins were expressed using the Champion pET151/D-TOPO protein expression system (Invitrogen, Thermo Scientific, St. Leon-Roth, Germany). Expression and purification of the wild type and mutated proteins were carried out as described previously^21^. Briefly, *E. coli* BL21 (DE3) was used for the overexpression of His6-tagged proteins. Overexpressed proteins were obtained after a pre-incubation of 150 mL of bacterial culture at 37 °C until OD_600_ of 0.8 -1 was reached. Gene expression was induced with 0.5 mM isopropylthio-β-galactoside (Carl Roth, Karlsruhe, Germany) and incubated for 20 h at 20 °C. Crude extracts were obtained by incubating the cell pellet with lysozyme (final concentration, 1 mg/mL), sonication, and centrifugation to separate cell debris from the protein-containing soluble fraction. The overexpressed protein was purified by Ni–NTA affinity chromatography (Qiagen, Hilden, Germany) according to the manufacturer’s instructions. Protein concentrations were measured using the standard Bradford assay^75^. Protein purity was confirmed using SDS-PAGE (Fig. S18). The purified proteins were stored at − 20 °C or − 70 °C for further use.

### Enzyme assay

Double enzyme assays, to determine sodorifen formation, were performed using FPPMT wildtype or mutated enzyme together with the SODS enzyme. The reaction tubes containing 20 μg of each purified enzyme, 50 μL assay buffer (250 mM HEPES–KOH, 100 mM MgCl_2_, 2.5 mM MnCl_2_, 50% (v/v) glycerol, pH 8), 30 mM dithiothreitol, 2.3 mM of S-adenosyl methionine (Merck Sigma-Aldrich, Darmstadt, Germany), 0.06 mM of farnesyl pyrophosphate (Echelon Biosciences, Salt Lake City, USA) and double distilled water (ad 200 μL) were incubated at 37 °C for 3 h 30 min. To determine pre-sodorifen synthesis, coupled enzyme assays were performed as described above (for the double enzyme assays) by starting the reaction with the FPPMT wildtype or mutant enzyme (without SODS (terpene synthase)). After incubation at 37 °C for 3 h 30 min, 10 U of alkaline phosphatase (Thermo Scientific, St. Leon-Roth, Germany) was added to the reaction mix and incubated for 1 h at 37 °C. Subsequently, each enzyme assay was overlaid with 200 μL hexane (containing 5 ng/μL nonyl acetate as internal standard). The reaction products were extracted by vortexing for 30 s followed by centrifugation (2 min at 5000*g*). The top layer representing the hexane phase was removed for GC–MS analysis. For the analysis of pre-sodorifen pyrophosphate, enzyme assays were performed as described above except that FPP was incubated only with FPPMT at 37 °C for 3 h 30 min. Thereafter, proteins in the reaction mix were precipitated by the addition of 50% (v/v) acetonitrile (Carl Roth, Karlsruhe, Germany) and the reaction mixture was filtered using 10 kDa molecular-mass cut-off Amicon Ultra filter (Merck Millipore, Darmstadt, Germany). The filtrate was lyophilized reconstituted in 700 µL of acetonitrile/water 7:3 and analyzed by LC–MS.

### GC–MS analysis

The volatile compounds were analyzed with a Shimadzu GC–MS-QP500 or QP2010 system (Kyoto, Japan) with a CTC autosampler (CTC Analytics, Zwingen, Switzerland) equipped with a DB5-MS column (60 m × 0.25 mm × 0.25 μm; J&W Scientific, Folsom, California, USA). Samples of 1 μL were injected at 200 °C using splitless mode. Helium was used as carrier gas at a flow rate of 1.1 mL/min. A temperature gradient was applied by starting from 35 °C for 2 min followed by an increase of 10 °C/min to 280 °C within 24.5 min, followed by 15 min at 280 °C. Electron ionization at 70 eV was used. Mass spectra were obtained using the scan mode (with *m/z* 40–280). Data were analyzed using the Lab Solution software (Shimadzu, Duisburg, Germany). Compound identity was confirmed by comparison of the mass spectra and GC retention times with those of sodorifen and pre-sodorifen.

### LC–MS analysis

LC–MS analysis was performed using a Nexera X2 liquid chromatograph (Shimadzu Corporation, Kyoto, Japan) coupled to an AB Sciex QTRAP 5500 mass spectrometer (AB Sciex GmbH, Darmstadt Germany). Data were analyzed using the Analyst Instrument and Data Processing Software Version 1.6.3. Proteins in the enzyme assay reaction mix were precipitated by the addition of 50% acetonitrile and ultra-filtrated. FPP, PSPP, and SAM were separated by ion-pair chromatography according to Balcke et al.^76^. Briefly, the samples were separated on a Nucleoshel RP18, 2.7 µm column (150 × 2 mm) (Macherey Nagel, Düren, Germany) with a linear gradient of 10 mM aqueous tributylamine (eluent A) adjusted to pH 6.2 with acetic acid and acetonitrile (eluent B) at a flow rate of 0.4 ml/min. General MS parameters: negative ionization (− 4.5 kV), source temperature 450 °C. Mass transitions and compound dependent parameters for SAM and FPP were taken from^76^. Mass transition and compound dependent parameters for PSPP were determined by direct infusion of the reaction mix into the MS. The mass transition of the putative PSPP (*m/z* 395.1) to the most prominent product ion (phosphate (*m/z* 78.8)) was optimized. In MRM mode the following compound dependent parameters were used for SAM (mass transition: 356.0/133.9 Da), FPP (mass transition 381.3/78.9), and PSPP (mass transition 395.1/78.8). The declustering potential was − 40, − 50, − 40 V, the collision energy at − 24, − 50, − 40 V, and collision exit potential at − 7, − 5, − 3 V, respectively.

## Supplementary Information

Supplementary Information 1.

Supplementary Information 2.

## References

[1] Buckingham J, Cooper C, Purchase R (2016). Natural products desk reference.

[2] Christianson DW (2017). Structural and Chemical Biology of Terpenoid Cyclases. Chem. Rev..

[3] Goldstein JL, Brown MS (1990). Regulation of the mevalonate pathway. Nature.

[4] Eisenreich W (1998). The deoxyxylulose phosphate pathway of terpenoid biosynthesis in plants and microorganisms. Chem. Biol..

[5] Dickschat JS (2007). Biosynthesis of the Off-Flavor 2-Methylisoborneol by the Myxobacterium Nannocystis exedens. Angew. Chem. Int. Ed..

[6] Komatsu M, Tsuda M, Ōmura S, Oikawa H, Ikeda H (2008). Identification and functional analysis of genes controlling biosynthesis of 2-methylisoborneol. Proc. Natl. Acad. Sci..

[7] Wang C-M, Cane DE (2008). Biochemistry and molecular genetics of the biosynthesis of the earthy odorant methylisoborneol in *Streptomyces coelicolor*. J. Am. Chem. Soc..

[8] Ignea C (2018). Synthesis of 11-carbon terpenoids in yeast using protein and metabolic engineering. Nat. Chem. Biol..

[9] Kschowak MJ, Wortmann H, Dickschat JS, Schrader J, Buchhaupt M (2018). Heterologous expression of 2-methylisoborneol / 2 methylenebornane biosynthesis genes in *Escherichia coli* yields novel C11-terpenes. PLoS ONE.

[10] Drummond L (2019). Expanding the Isoprenoid Building Block Repertoire with an IPP Methyltransferase from *Streptomyces monomycini*. ACS Synth. Biol..

[11] Ishiyama A (2008). In vitro and in vivo antitrypanosomal activitiy of two microbial metabolites, KS-505a and Alazopeptin. J. Antibiot. (Tokyo).

[12] Ozaki T (2018). Enzymatic formation of a skipped methyl-substituted octaprenyl side chain of longestin (KS-505a): Involvement of homo-IPP as a common extender unit. Angew. Chem. Int. Ed..

[13] Eiben CB (2019). Mevalonate Pathway Promiscuity Enables Noncanonical Terpene Production. ACS Synth. Biol..

[14] Kai M (2010). *Serratia odorifera*: analysis of volatile emission and biological impact of volatile compounds on *Arabidopsis thaliana*. Appl. Microbiol. Biotechnol..

[15] von Reuß, S. H., Kai, M., Piechulla, B. & Francke, W. Octamethylbicyclo[3.2.1]octadienes from the Rhizobacterium *Serratia odorifera*. *Angew. Chem. Int. Ed.* **49**, 2009–2010 (2010). 10.1002/anie.200905680 20155769

[16] Weise T (2014). VOC emission of various *Serratia* species and isolates and genome analysis of *Serratia plymuthica* 4Rx13. FEMS Microbiol. Lett..

[17] Schmidt, R. *et al.* Fungal volatile compounds induce production of the secondary metabolite Sodorifen in *Serratia plymuthica* PRI-2C. *Sci. Rep.* **7**, (2017). 10.1038/s41598-017-00893-3 PMC5429845 28408760

[18] Kai, M. & Piechulla, B. Interspecies interaction of *Serratia plymuthica* 4Rx13 and Bacillus subtilis B2g alters the emission of sodorifen. *FEMS Microbiol. Lett.* **365**, (2018). 10.1093/femsle/fny253 30307482

[19] Domik, D., Magnus, N. & Piechulla, B. Analysis of a new cluster of genes involved in the synthesis of the unique volatile organic compound sodorifen of *Serratia plymuthica* 4Rx13. *FEMS Microbiol. Lett.* **363**, (2016). 10.1093/femsle/fnw139 27231241

[20] Domik, D. *et al.* A Terpene Synthase Is Involved in the Synthesis of the Volatile Organic Compound Sodorifen of *Serratia plymuthica* 4Rx13. *Front. Microbiol.* **7**, (2016). 10.3389/fmicb.2016.00737 PMC4872519 27242752

[21] von Reuss S (2018). Sodorifen biosynthesis in the Rhizobacterium *Serratia plymuthica* involves methylation and cyclization of mep-derived farnesyl pyrophosphate by a SAM-dependent C-methyltransferase. J. Am. Chem. Soc..

[22] Human Metabolome Database: Showing metabocard for Farnesyl pyrophosphate (HMDB0000961). https://hmdb.ca/metabolites/HMDB0000961.

[23] Robetta Server. http://www.robetta.org/submit.jsp. http://www.robetta.org/submit.jsp.

[24] Protein Modelle: Leibniz-Institut für Pflanzenbiochemie. https://www.ipb-halle.de/infrastruktur/datenbanken-und-tools/protein-modelle/?MP=6-1058https://www.ipb-halle.de/infrastruktur/datenbanken-und-tools/protein-modelle/?MP=6-1058 (2020).

[25] Stewart, J. J. P. MOPAC2016, Stewart Computational Chemistry, Colorado Springs. http://openmopac.net/MOPAC2016.html (2016).

[26] Brandt W, Manke K, Vogt T (2015). A catalytic triad—Lys-Asn-Asp—Is essential for the catalysis of the methyl transfer in plant cation-dependent O-methyltransferases. Phytochemistry.

[27] Tantillo DJ (2011). Biosynthesis via carbocations: Theoretical studies on terpene formation. Nat. Prod. Rep..

[28] Raz K, Levi S, Gupta PK, Major DT (2020). Enzymatic control of product distribution in terpene synthases: insights from multiscale simulations. Curr. Opin. Biotechnol..

[29] Lee SG, Kim Y, Alpert TD, Nagata A, Jez JM (2012). Structure and Reaction Mechanism of Phosphoethanolamine Methyltransferase from the Malaria Parasite *Plasmodium falciparum*. J. Biol. Chem..

[30] Saen-oon S, Lee SG, Jez JM, Guallar V (2014). An Alternative Mechanism for the Methylation of Phosphoethanolamine Catalyzed by *Plasmodium falciparum* Phosphoethanolamine Methyltransferase. J. Biol. Chem..

[31] Awakawa T (2014). A methyltransferase initiates terpene cyclization in teleocidin B biosynthesis. J. Am. Chem. Soc..

[32] Yu F (2016). Crystal structure and enantioselectivity of terpene cyclization in SAM-dependent methyltransferase TleD. Biochem. J..

[33] Starks CM, Back K, Chappell J, Noel JP (1997). Structural basis for cyclic terpene biosynthesis by tobacco 5-Epi-aristolochene synthase. Science.

[34] Thoma R (2004). Insight into steroid scaffold formation from the structure of human oxidosqualene cyclase. Nature.

[35] Köksal M, Chou WKW, Cane DE, Christianson DW (2012). Structure of geranyl diphosphate C-methyltransferase from *Streptomyces coelicolor* and implications for the mechanism of isoprenoid modification. Biochemistry.

[36] Gurowietz, A. (2017) Investigation of putative catalytic mechanisms of a farnesyl-methyltransferase from *Serratia plymuthica*. Master thesis, The Martin Luther University Halle-Wittenberg.

[37] Martin JL, McMillan FM (2002). SAM (dependent) I AM: the S-adenosylmethionine-dependent methyltransferase fold. Curr. Opin. Struct. Biol..

[38] Vidgren J, Svensson LA, Liljas A (1994). Crystal structure of catechol O -methyltransferase. Nature.

[39] Cheng X, Kumar S, Posfai J, Pflugrath JW, Roberts RJ (1993). Crystal structure of the Hhal DNA methyltransferase complexed with S-adenosyl-l-methionine. Cell.

[40] Liscombe DK, Louie GV, Noel JP (2012). Architectures, mechanisms and molecular evolution of natural product methyltransferases. Nat. Prod. Rep..

[41] Schubert HL, Blumenthal RM, Cheng X (2003). Many paths to methyltransfer: a chronicle of convergence. Trends Biochem. Sci..

[42] Struck A-W, Thompson ML, Wong LS, Micklefield J (2012). S-adenosyl-methionine-dependent methyltransferases: highly versatile enzymes in biocatalysis, biosynthesis and other biotechnological applications. Chembiochem Eur. J. Chem. Biol..

[43] Schluckebier G, O’Gara M, Saenger W, Cheng X (1995). Universal Catalytic Domain Structure of AdoMet-dependent Methyltransferases. J. Mol. Biol..

[44] Hou, A., Lauterbach, L. & Dickschat, J. S. Enzymatic synthesis of methylated terpene analogues using the plasticity of bacterial terpene synthases. *Chem. Eur. J.* **26**, 2178–2182 (2020). 10.1002/chem.201905827 PMC7065205 31898827

[45] Torres MA (2016). Structural and functional studies of pavine N-Methyltransferase from *Thalictrum flavum* reveal novel insights into substrate recognition and catalytic mechanism. J. Biol. Chem..

[46] Abeykoon AH (2016). Structural Insights into Substrate Recognition and Catalysis in Outer Membrane Protein B (OmpB) by Protein-lysine Methyltransferases from *Rickettsia*. J. Biol. Chem..

[47] Syed Ibrahim, B., Burley, S. K. & Swaminathan, S. Crystal Structure of Methyl Transferase from *Methanosarcina mazei*. *BE Publ.*10.2210/pdb3mgg/pdb.

[48] Krieger E (2009). Improving physical realism, stereochemistry and side-chain accuracy in homology modeling: four approaches that performed well in CASP8. Proteins.

[49] Krieger E, Vriend G (2014). YASARA View—molecular graphics for all devices—from smartphones to workstations. Bioinformatics.

[50] Krieger E, Vriend G (2015). New ways to boost molecular dynamics simulations. J. Comput. Chem..

[51] Sippl MJ (1990). Calculation of conformational ensembles from potentials of mena force: An approach to the knowledge-based prediction of local structures in globular proteins. J. Mol. Biol..

[52] Sippl MJ (1993). Recognition of errors in three-dimensional structures of proteins. Proteins Struct. Funct. Bioinforma..

[53] Laskowski RA, MacArthur MW, Moss DS, Thornton JM (1993). PROCHECK: a program to check the stereochemical quality of protein structures. J. Appl. Crystallogr..

[54] Verdonk ML, Cole JC, Hartshorn MJ, Murray CW, Taylor RD (2003). Improved protein–ligand docking using GOLD. Proteins Struct. Funct. Bioinforma..

[55] Hartshorn MJ (2007). Diverse, high-quality test set for the validation of protein−ligand docking performance. J. Med. Chem..

[56] Korb O, Stützle T, Exner TE (2009). Empirical scoring functions for advanced protein−ligand docking with PLANTS. J. Chem. Inf. Model..

[57] Korb O (2012). Potential and limitations of ensemble docking. J. Chem. Inf. Model..

[58] Gerber PR, Müller K (1995). MAB, a generally applicable molecular force field for structure modelling in medicinal chemistry. J. Comput. Aided Mol. Des..

[59] Case DA (2014). Amber 14 Reference Manual.

[60] Stewart JJP (2013). Optimization of parameters for semiempirical methods VI: more modifications to the NDDO approximations and re-optimization of parameters. J. Mol. Model..

[61] Broyden CG (1970). The Convergence of a Class of Double-rank Minimization Algorithms 1 General Considerations. IMA J. Appl. Math..

[62] Fletcher R (1970). A new approach to variable metric algorithms. Comput. J..

[63] Goldfarb D (1970). A family of variable-metric methods derived by variational means. Math. Comput..

[64] Shanno, D. F. Conditioning of quasi-newton methods for function minimization. 10.

[65] Stewart Computational Chemistry—MOPAC Home Page. *Basic Molecular Tools, MOE GUI for MOPAC2012 scan/grid calculations* http://svl.chemcomp.com/filedetails.php?lid=1004&cid=37.

[66] PyMOL | pymol.org. https://pymol.org/2/.

[67] Neese, F.; Becker, U.; Ganyushin, D.; Hansen, A.; Liakos, D.; Kollmar, C.; Koßmann, S.; Petrenko, T.; Reimann, C.; Riplinger, C.; Sivalingam, K.; Wezisla, B.; Wennmohs, F. Orca Download. *ORCA - an ab initio, density functional and semiempirical program package* https://cec.mpg.de/orcadownload/.

[68] Becke A (1988). Density-functional exchange-energy approximation with correct asymptotic behavior. Phys. Rev. Gen. Phys..

[69] Karton A, Tarnopolsky A, Lamère J-F, Schatz GC, Martin JML (2008). J. Phys. Chem. A.

[70] Schäfer A, Horn H, Ahlrichs R (1992). Fully optimized contracted Gaussian basis sets for atoms Li to Kr. J. Chem. Phys..

[71] Weigend F, Ahlrichs R (2005). Balanced basis sets of split valence, triple zeta valence and quadruple zeta valence quality for H to Rn: Design and assessment of accuracy. Phys. Chem. Chem. Phys..

[72] Neese F (2012). The ORCA program system. WIREs Comput. Mol. Sci..

[73] Sinnecker S, Rajendran A, Klamt A, Diedenhofen M, Neese F (2006). Calculation of solvent shifts on electronic g-tensors with the conductor-like screening model (COSMO) and its self-consistent generalization to real solvents (Direct COSMO-RS). J. Phys. Chem. A.

[74] Bruhn T, Schaumlöffel A, Hemberger Y, Bringmann G (2013). SpecDis: quantifying the comparison of calculated and experimental electronic circular dichroism spectra. Chirality.

[75] Bradford MM (1976). A rapid and sensitive method for the quantitation of microgram quantities of protein utilizing the principle of protein-dye binding. Anal. Biochem..

[76] Balcke, G. U., Bennewitz, S., Zabel, S. & Tissier, A. Isoprenoid and Metabolite Profiling of Plant Trichomes. in *Plant Isoprenoids: Methods and Protocols* (ed. Rodríguez-Concepción, M.) 189–202 (Springer, 2014). 10.1007/978-1-4939-0606-2_13. 10.1007/978-1-4939-0606-2_13 24777798

[77] BLAST. https://www.uniprot.org/blast/.

[78] Multalin interface page. http://multalin.toulouse.inra.fr/multalin/.

[79] Kozbial PZ, Mushegian AR (2005). Natural history of S-adenosylmethionine-binding proteins. BMC Struct. Biol..

[80] Cheng X (1995). Structure and Function of DNA Methyltransferases. Annu. Rev. Biophys. Biomol. Struct..

