# Supplementary material for: Reaction mechanism of the farnesyl pyrophosphate C-methyltransferase towards the biosynthesis of pre-sodorifen pyrophosphate by Serratia plymuthica 4Rx13
Source: Sci Rep. 2021 Feb 4;11:3182. doi: 10.1038/s41598-021-82521-9 (PMC7862628; doi:10.1038/s41598-021-82521-9)
Supplement: Supplementary file 2 — Supplementary Information 2. [file 41598_2021_82521_MOESM2_ESM.docx]

**Supplementary information**

**Reaction mechanism of the farnesyl pyrophosphate *C*-methyltransferase towards the synthesis of pre-sodorifen pyrophosphate by *Serratia plymuthica* 4Rx13**

Marie Chantal Lemfack^#^*^1^, Wolfgang Brandt^#^**^2^, Katja Krüger^1,5^, Alexandra Gurowietz^2,6^, Jacky Djifack^1,7^, Jan-Philip Jung^1^, Marius Hopf^1,8^, Heiko Noack^3^, Björn Junker^3^, Stephan von Reuß^4^, Birgit Piechulla^1^

^1^Institute of Biological Sciences, University of Rostock, Albert-Einstein-Straße 3, D-18059 Rostock, Germany

^2^Department of Bioorganic Chemistry, Leibniz-Institute of Plant Biochemistry, Weinberg 3, D-06120 Halle, Germany

^3^Institute of Pharmacy/Biosynthesis of Active Substances, Hoher Weg 8, D-06120 Halle (Saale), Germany

^4^Laboratory of Bioanalytical Chemistry, Institute of Chemistry, University of Neuchatel, Avenue de Bellevaux 51, CH-2000 Neuchâtel, Switzerland

^5^Department of Internal Medicine I, University Hospital RWTH Aachen, 52074 Aachen, Germany

^6^Institute of Biology, Martin-Luther-Universität Halle-Wittenberg, Weinberg 10, D-06120 Halle (Saale), Germany

^7^PIMAN Consultants, 12 Rue Barthelemy Danjou, 92100 Boulogne-Billancourt, France

^8^Duale Hochschule Gera-Eisenach, Weg der Freundschaft 4, D-07546 Gera, Germany

^#^Equal contribution

*corresponding author

**List of the material included**

Table S1: list of all the primers used for the site directed mutagenesis

Table S2: Results of DFT calculations

Table S3: GC-MS analysis of the enzyme assay products

Figure S1: Fragmentation pattern of the putative pre-sodorifen pyrophosphate (PSPP) in the crude enzyme assay

Figure S2: Fragmentation patterns of PSPP and FPP

Figure S3: Prosa II plot of the knowledge-based energy in dependence of the amino acid sequence of the *Serratia plymutica* FPP *C*-methyltransferase

Figure S4: Ramachandran plot of the model of the *Serratia plymuthica* FPP *C*-methyltransferase

Figure S5: Energy profile of the entire reaction. Numbers correspond to the reaction steps and structures shown in figure 5

Figure S6: Model used for the DFT-calculations

Figure S7: The outer space of the binding pocket of the *Serratia plymuthica* FPP *C*-methyltransferase showing all amino acid residues that were mutated to alanine

Figure S8: TIC chromatograms after GC-MS analysis of the products of the *Serratia plymuthica* FPP *C*-methyltransferase mutant enzymes in comparison to the wildtype

Figure S9: TIC chromatograms after GC-MS analysis of the products of the *Serratia plymuthica* FPP *C*-methyltransferase mutant enzymes in comparison to the wildtype

Figure S10: TIC chromatograms of GC-MS analysis of the products of the *Serratia plymuthica* FPP *C*-methyltransferase mutant in comparison to the wildtype enzyme

Figure S11: TIC chromatograms after GC-MS analysis of the products of the *Serratia plymuthica* FPP *C*-methyltransferase mutant enzymes in comparison to the wildtype

Figure S12: TIC chromatograms of GC-MS analysis of the products of the H45A, E297A, L239A, and C241A mutant FPP methyltransferase in comparison to the wildtype enzyme

Figure S13: TIC chromatograms of GC-MS analysis of the products of the H45A, E297A, L239A, and C241A mutant FPP methyltransferase in comparison to the wildtype enzyme

Figure S14: Sequence alignment (BLOSUM62) of 14 annotated microbial methyltransferases

Figure S15: Mass spectra of compound #1, #2 and #3

Figure S16: Mass spectra of compound #4, #5 and #6

Figure S17: Superposition of the SAH binding site of 5KOK (grey backbone secondary structure) with the model of the *Serratia plymutica* FPP *C*-methyltransferase (green)

Figure S18: Documentation of the SDS-PAGE gels of IPTG induced samples of *E. coli* BL21(DE3) cultures of the wild type and all mutant of *Serratia plymuthica* FPP *C*-methyltransferase

**Tables**

**Table S2: Results of DFT calculations.**

| **Step*** | energies  (in hartree) | energies  (in kcal/mol) | relative energies  (in kcal/mol) | relative energies##  (in kcal/mol) |
| --- | --- | --- | --- | --- |
| **1** | -4223.7416 | -2650397.9 | 0.0 | 0.0 |
| **1#** | -4223.7155 | -2650381.5 | 16.4 | 16.4 |
| **2** | -4223.7502 | -2650403.2 | -5.3 | -5.3 |
| **2#** | -4223.7262 | -2650388.2 | 9.7 | 15.0 |
| **3** | -4223.7487 | -2650402.3 | -4.4 | 0.9 |
| **3#** | -4223.7241 | -2650386.9 | 11.0 | 15.9 |
| **4** | -4223.7511 | -2650403.8 | -5.9 | -1.5 |
| **4#** | -4223.6996 | -2650371.5 | 26.3 | 32.3 |
| **5** | -4223.7487 | -2650402.3 | -4.4 | 1.5 |
| **5#** | -4223.7303 | -2650390.8 | 7.1 | 11.5 |
| **6** | -4223.7445 | -2650399.7 | -1.8 | 2.6 |
| **6#** | -4223.7243 | -2650387.0 | 10.9 | 12.7 |
| **7** | -4223.7429 | -2650398.7 | -0.8 | 1.0 |
| **7#** | -4223.7094 | -2650377.6 | 20.3 | 21.1 |
| **8** | -4223.7726 | -2650417.3 | -19.4 | -18.6 |

* Steps corresponding to the structures shown in Fig. 5. # indicates the transition state energies between the local intermediates. ## indicate the relative energies with regard to each previous stable intermediate like applied and discussed for Fig. 5.

**Table S3:** **GC-MS analysis of the enzyme assay products**. Double (for sodorifen production) or coupled enzyme assays (for pre-sodorifen) were performed with wildtype and mutant *Serratia plymuthica* FPP *C*-methyltransferase. Mutated amino acid residues are far away from the active site.

| **Sodorifen producer mutants** | | |
| --- | --- | --- |
| **Amino acids** | **mutants** | **Sodorifen/ sodorifen alcohol** |
| K34 | K34A | **Yes** |
| E41 | E41A | **Yes** |
| E44 | E44A | **Yes** |
| D63 | D63A | **Yes** |
| S190 | S190A | **Yes** |
| D218 | D218A | **Yes** |
| R242 | R242A | **Yes** |
| H275 | H275A | **Yes** |
| R286 | R286A | **Yes** |
| E298 | E298A | **Yes** |
| R300 | R300A | **Yes** |
| K312 | K312A | **Yes** |

**Figures and figure legends**


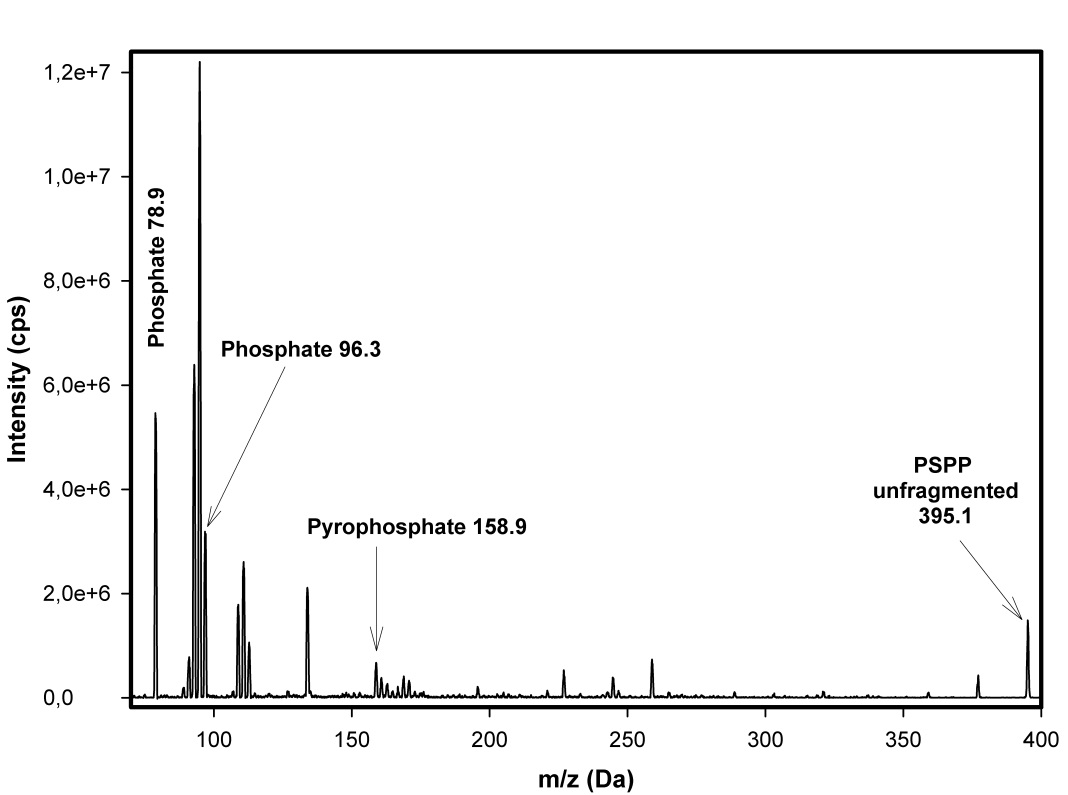


**Figure S1: Fragmentation pattern of the putative pre-sodorifen pyrophosphate (PSPP) in the crude enzyme assay.** FPP and SAM were incubated with FPP *C*-methyltransferase and directly infused into the MS. The mass of 395.1 Da (PSPP) was selected by Q1, fragmented with low collision energy, and scanned for product ions by Q3. The mass peak of 395.1 Da corresponds to PSPP. Phosphate and pyrophosphate fragments are also indicated.


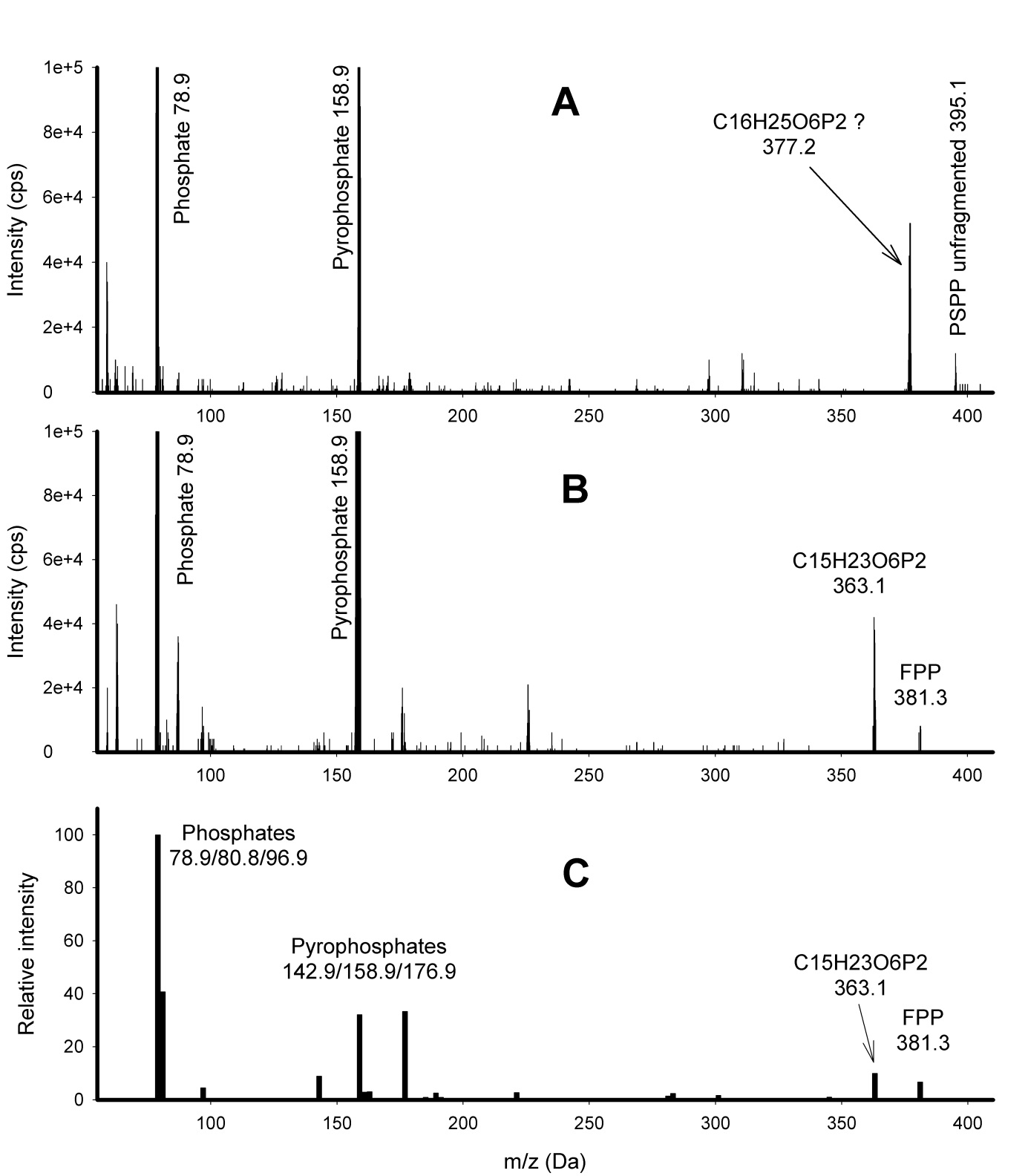


**Figure S2: Fragmentation patterns of PSPP and FPP.** The incubated reaction mix (SAM + FPP + *C*-methyltransferase) was separated by LC-MS and mass spectra were recorded for the peaks corresponding to PSPP (395.1 Da to x Da) **(A)** and FPP (381.3 Da to x Da) **(B)**. The major carbon fragment of PSPP has a mass of 377.2 Da, while the major carbon fragment of FPP has a mass of 363.1 Da (indicating an additional methylene group in the PSPP). (**C)** The fragmentation pattern of FPP was taken as reference from the Human Metabolome Database ^1^.





**Figure S3:** **Prosa II plot of the knowledge-based energy in dependence of the amino acid sequence of the *Serratia plymutica* FPP *C*-methyltransferase**. The entire sequence is in the negative energy region and the combined energy z-score of -10.15 indicated a native-like fold.


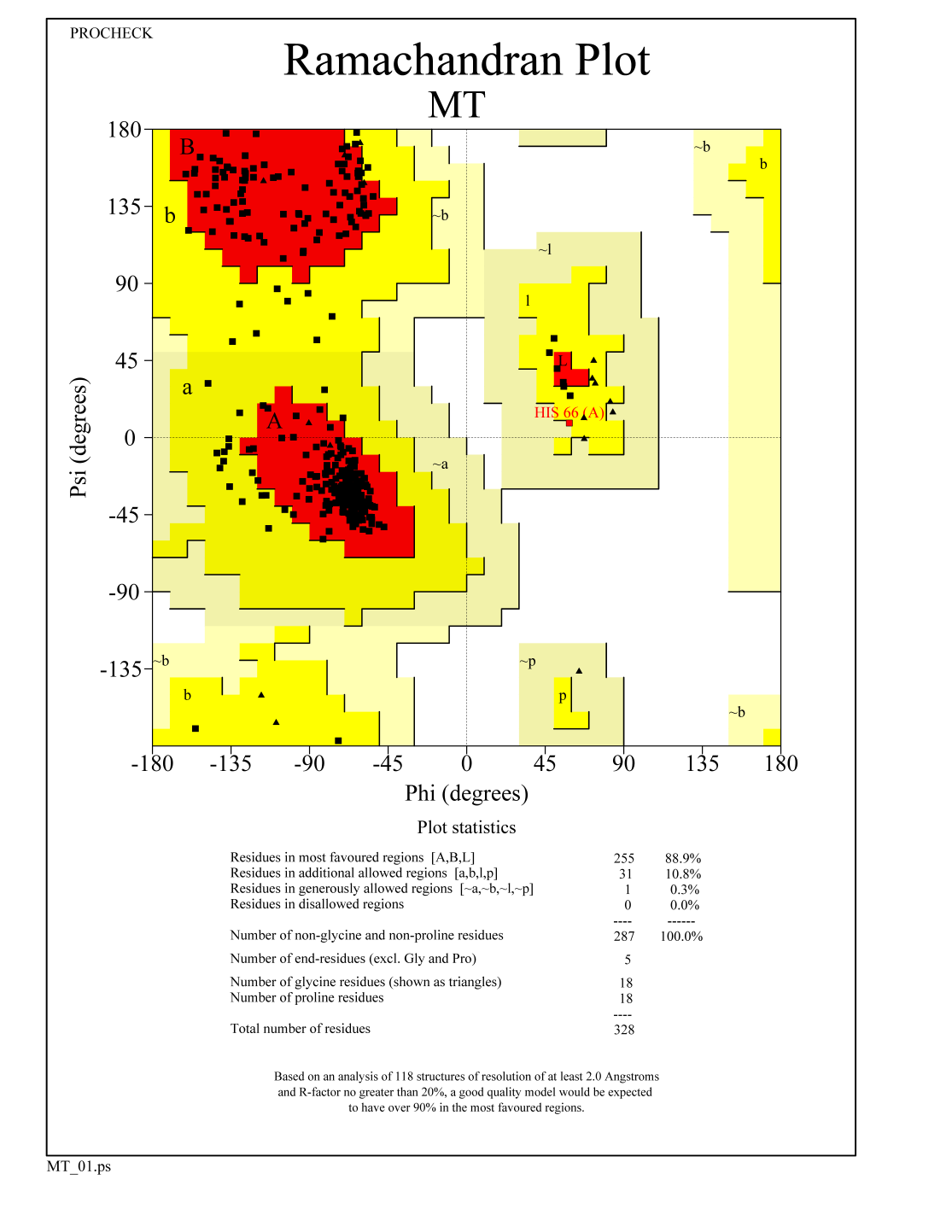


**Figure S4:** **Ramachandran plot of the model of the *Serratia plymuthica* FPP *C*-methyltransferase**. There are no outliers and all other stereochemical criterions are in the expected ranges.


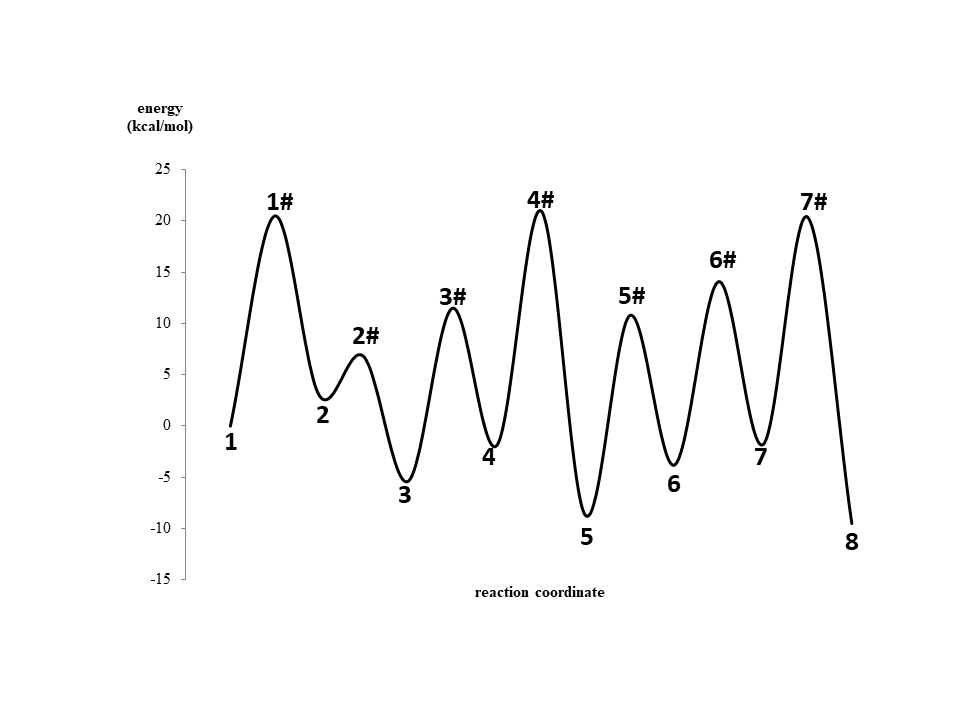


**Figure S5:** **Energy profile of the entire reaction. Numbers correspond to the reaction steps and structures shown in figure 5**. The transition states are characterized as follows: 1# distance C(SAM-methyl) - C10 =2.2 Å, 2# distance C6- C11 =2.2 Å, 3# distance C7- C9 =2.2 Å, 4# distance TyrOH-C8 =2.2 Å, 5# distance H6- C7 = 2.2 Å, 6# distance H11- C6 = 2.2 Å, 7# distance His-N- H = 2.2 Å.

**
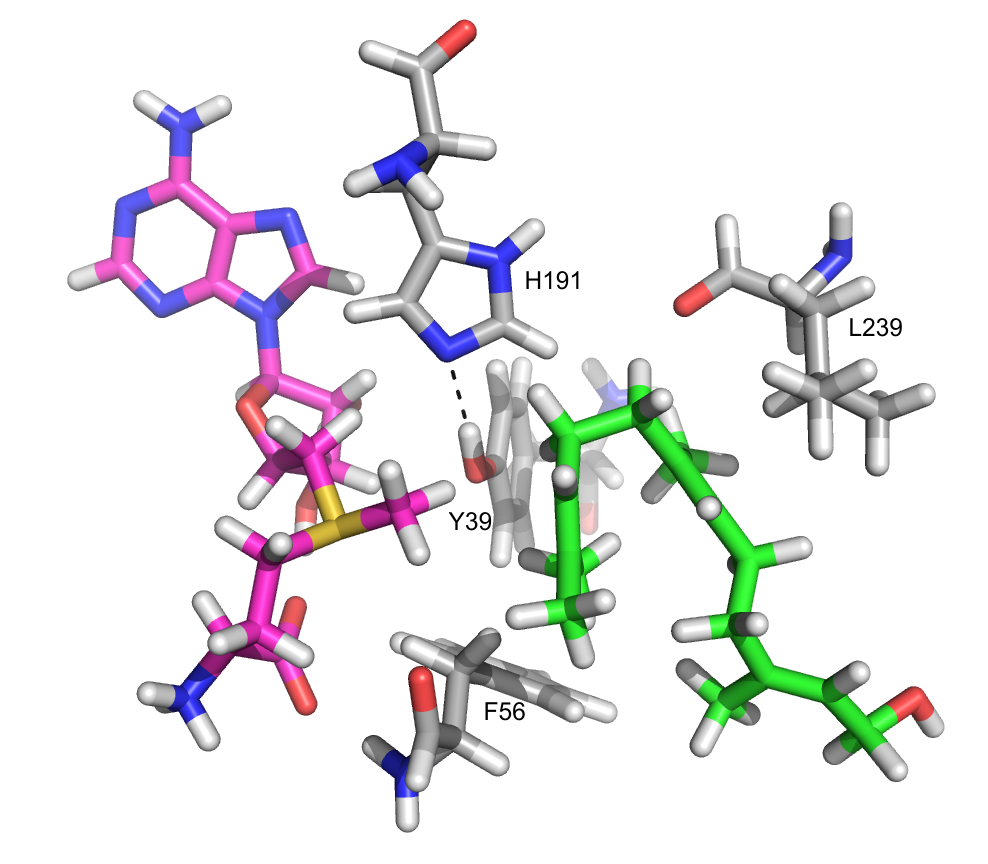
**

**Figure S6: Model that was used for the DFT-calculations.** All backbone atoms of F56, Y39, H191 and L239 were fixed during the energy optimization as well as the hydroxyl group of farnesyl alcohol (green carbon atoms) and parts of SAM (magenta carbon atoms).

**
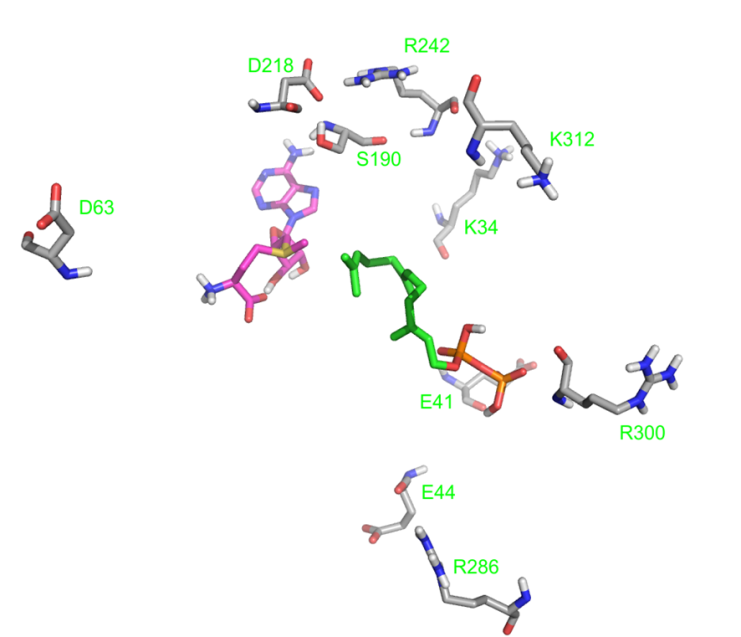
**

**Figure S7:** The outer space of the binding pocket of the *Serratia plymuthica* FPP *C*-methyltransferase showing all amino acid residues that were mutated to alanine but did not lead to inactivity or considerably reduced activity (Table S2). All these residues are rather far away from the binding of the ligands and therefore, mutation to alanine does not influence the catalytic activity essentially. SAM is shown in magenta, FPP in green.


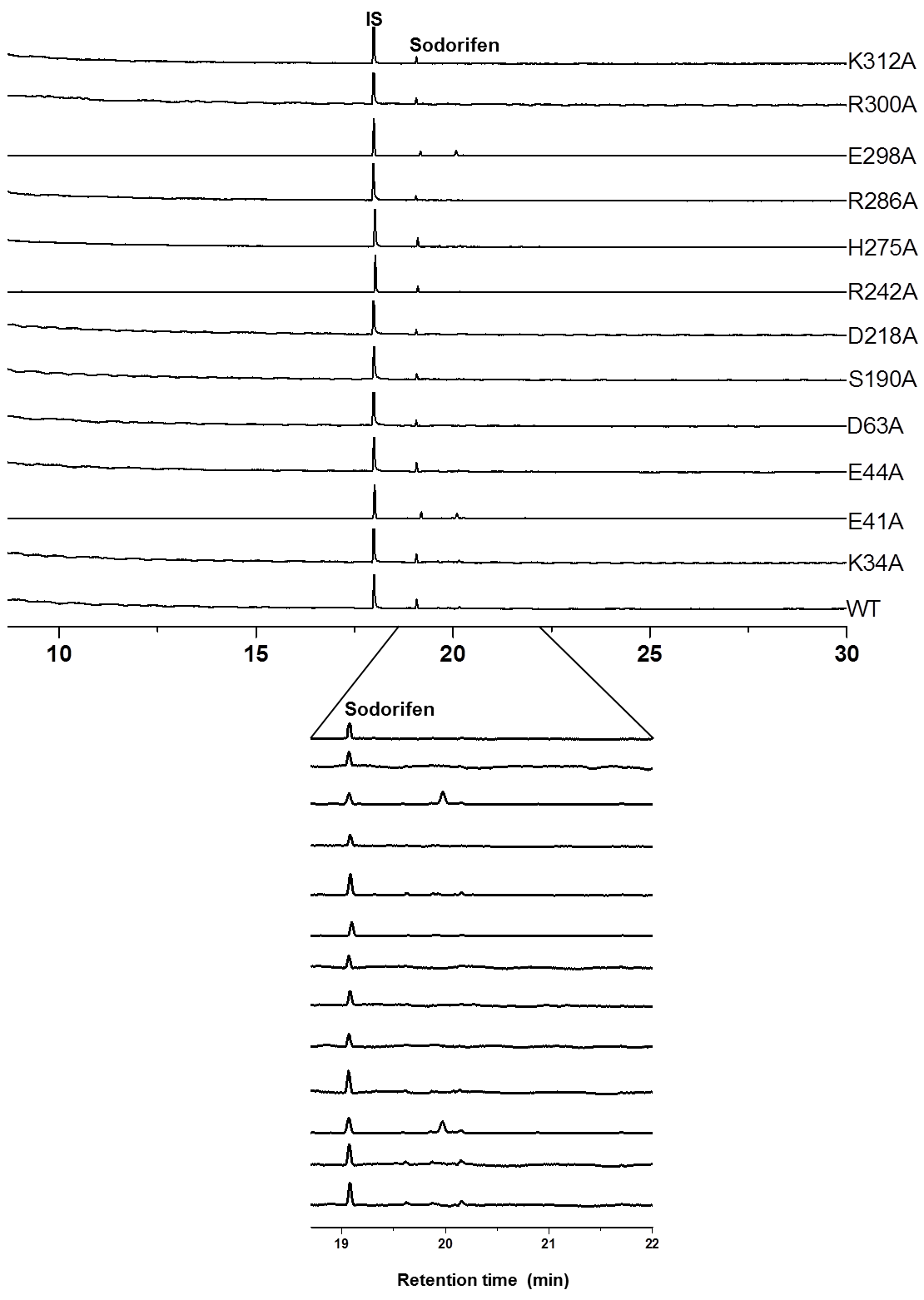


**Figure S8:** **TIC chromatograms after GC-MS analysis of the products of the *Serratia plymuthica* FPP *C*-methyltransferase mutant enzymes in comparison to the wildtype**. The mutated amino acids are located far from the active pocket. Mutated enzymes were analyzed in double enzyme assays (FPP was incubated together with the mutant or wildtype FPP methyltransferase and the *Serratia plymuthica* sodorifen terpene synthase (SODS)). Lower panel: enlarged view of the chromatogram from 18.5-22 min. IS: internal standard. All non-labeled peaks were also present in the control assay performed with the corresponding denatured enzyme. Number of replicates: n = 3-5.


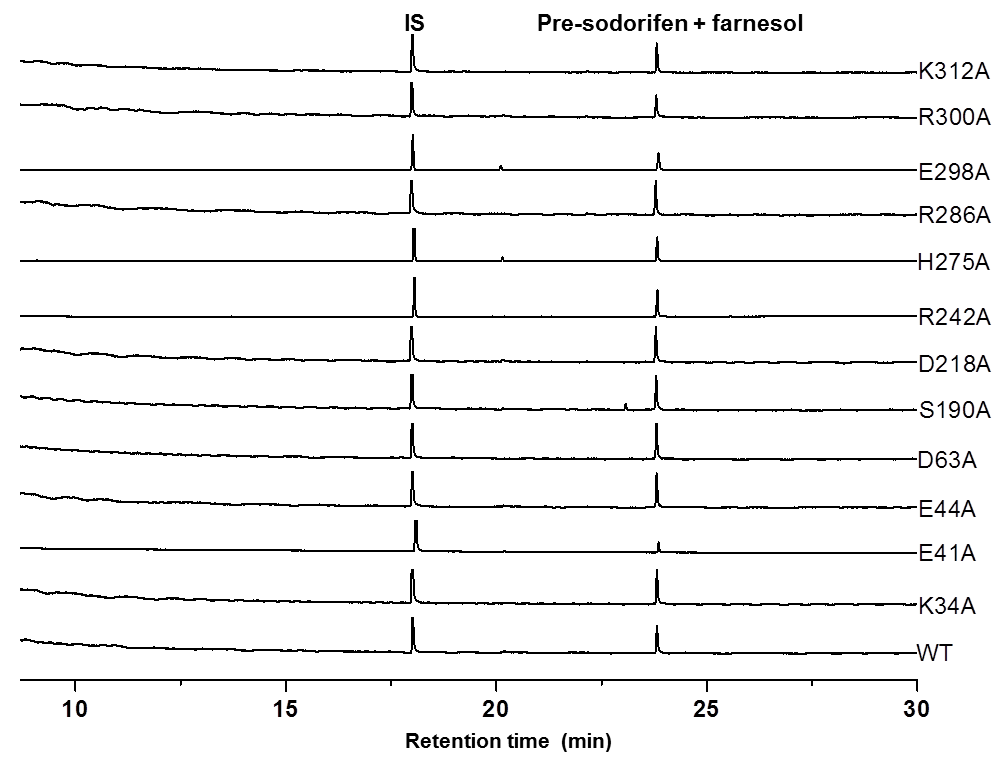


**Figure S9:** **TIC chromatograms after GC-MS analysis of the products of the *Serratia plymuthica* FPP *C*-methyltransferase mutant enzymes in comparison to the wildtype**. The mutated amino acids are far from the active pocket of the enzyme. Mutant enzymes were analyzed in coupled enzyme assays (FPP was incubated with the mutant or wildtype FPP methyltransferase and after the first incubation time the reaction mixture was subsequently incubated with alkaline phosphatase). IS: internal standard. Farnesol and pre-sodorifen have the same retention time. All non-labeled peaks were also present in the control assay performed with the corresponding denatured enzyme. Number of replicates: n = 3-5.


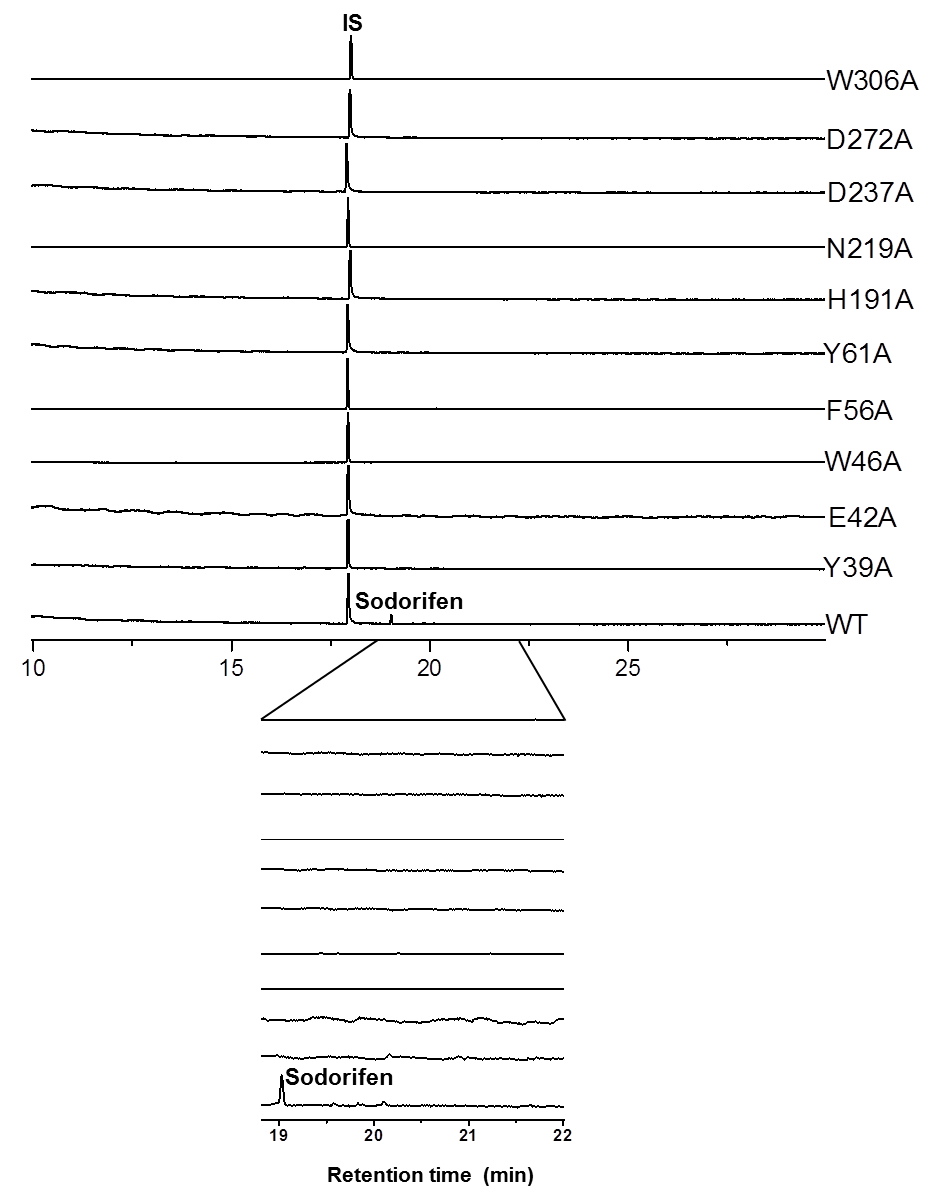


**Figure S10:** **TIC chromatograms of GC-MS analysis of the products of the *Serratia plymuthica* FPP *C*-methyltransferase mutant in comparison to the wildtype enzyme**. The mutated amino acids are located close or within the active pocket. Mutated enzymes were analyzed in double enzyme assays (FPP incubated together with mutant or wildtype FPP methyltransferase and sodorifen terpene synthase (SODS)). Lower panel: enlarged view of the chromatogram from 18.9-22 min. IS: internal standard. Number of replicates: n = 3-5.

**
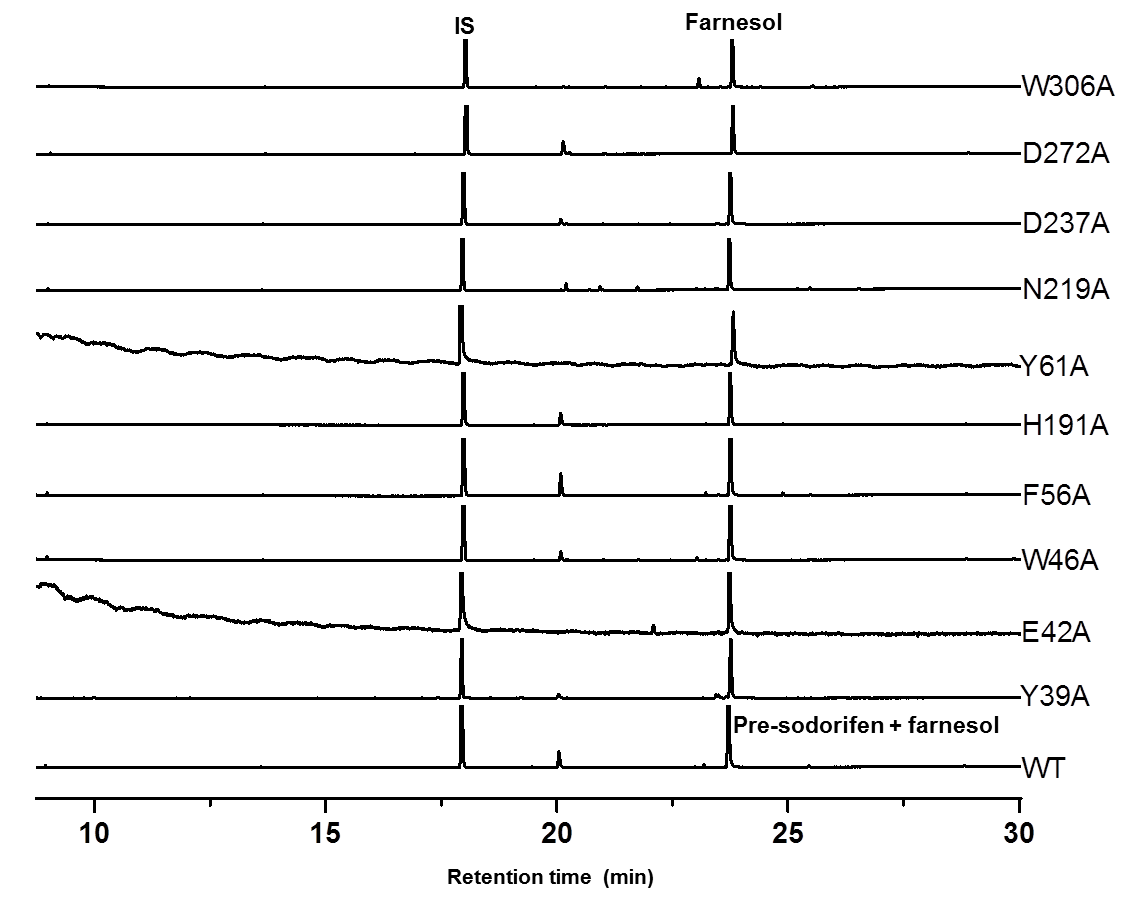
**

**Figure S11:** **TIC chromatograms after GC-MS analysis of the products of the *Serratia plymuthica* FPP *C*-methyltransferase mutant enzymes in comparison to the wildtype**. The mutated amino acids are close or within the active pocket. Mutated enzymes were analyzed in coupled enzyme assays (FPP was incubated with the mutant or wildtype FPP methyltransferase and after the first incubation time the reaction mixture was subsequently incubated with alkaline phosphatase). IS: internal standard. Farnesol and pre-sodorifen have the same retention time and are both present in the WT. All non-labeled peaks were also present in the control assay performed with the corresponding denatured enzyme. Number of replicates: n = 3-5.


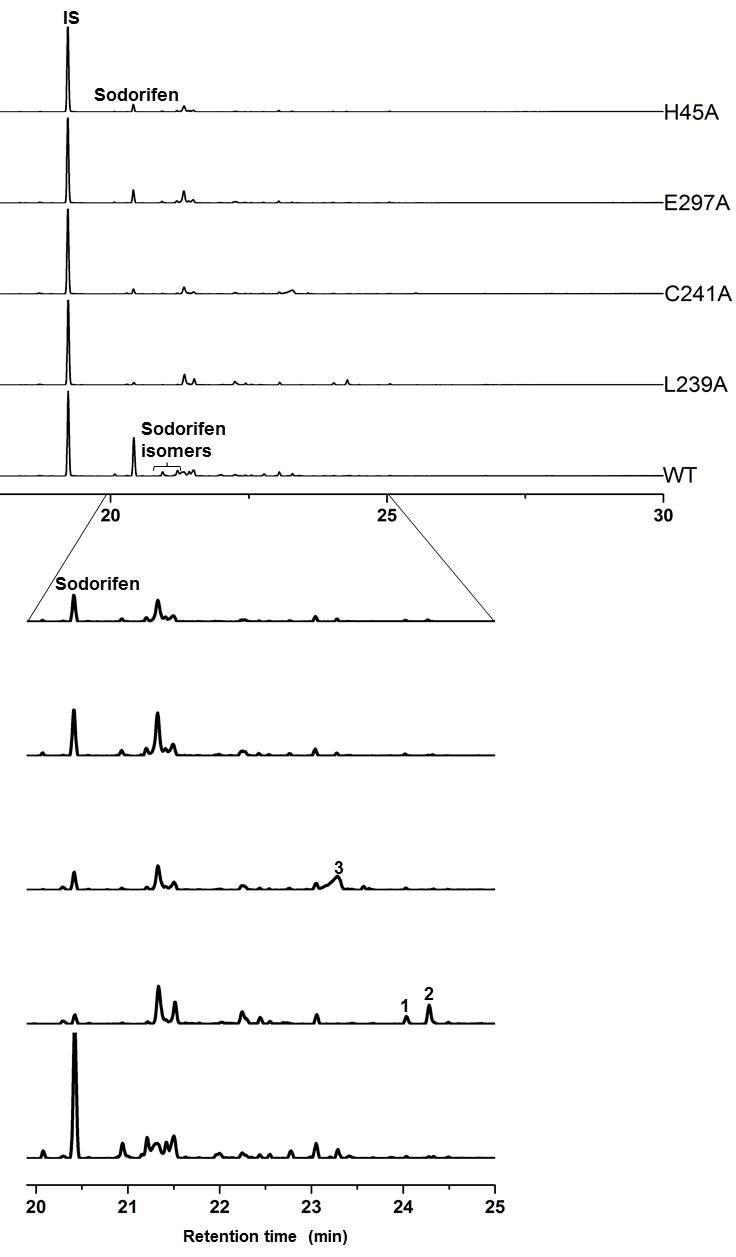


**Figure S12**: **TIC chromatograms of GC-MS analysis of the products of the H45A, E297A, L239A, and C241A mutant FPP methyltransferase in comparison to the wildtype enzyme**. The amino acids H45, E297, L239, and C241 are close or within the active pocket. Mutated enzymes were analyzed in double enzyme assays (FPP incubated with the mutant or wildtype FPP methyltransferase and sodorifen terpene synthase (SODS)). Lower panel: enlarged view of the chromatogram from 20 - 25 min. IS: Internal standard; #1, #2, and #3 designate new and yet unidentified compounds from L239A and C241A mutant enzymes. All non-labeled peaks were also present in the control assay performed with the corresponding denatured enzyme. Number of replicates: n = 3-5.


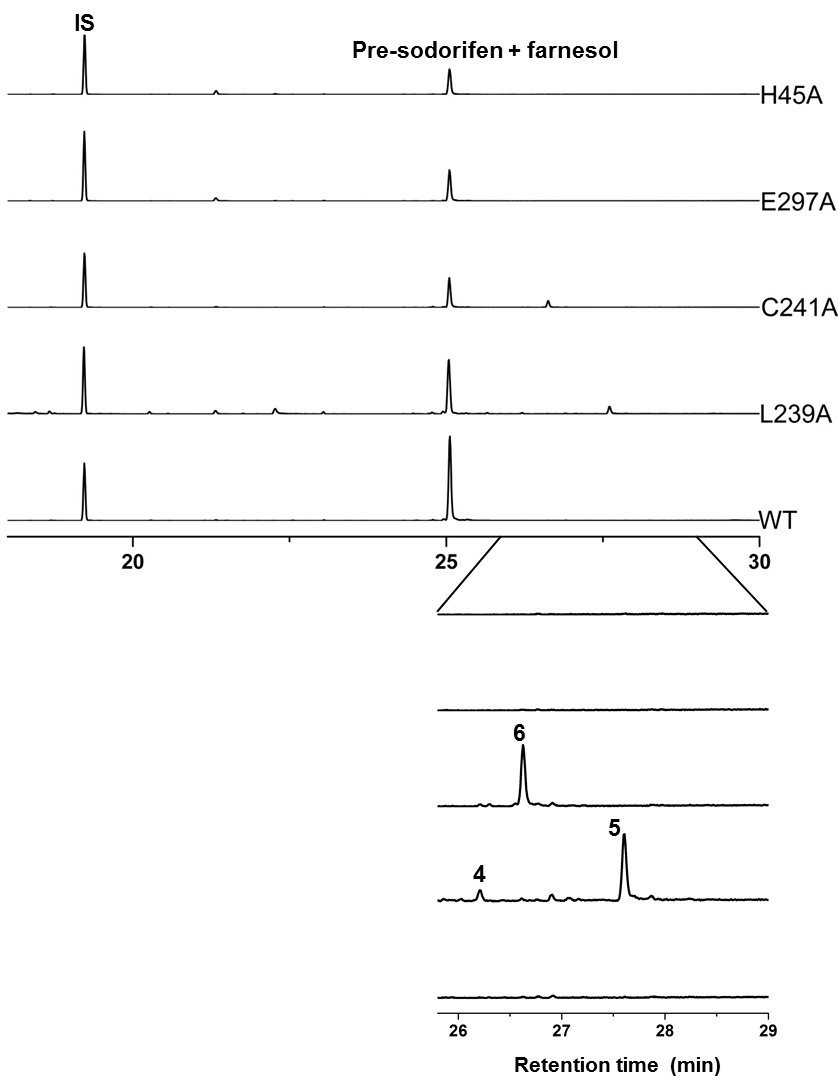


**Figure S13**: **TIC chromatograms of GC-MS analysis of the products of the H45A, E297A, L239A, and C241A mutant FPP methyltransferase in comparison to the wildtype enzyme**. The amino acids H45, E297, L239, and C241 are close or within the active pocket. Mutated enzymes were analyzed in coupled enzyme assays (FPP incubated with the mutant or wildtype FPP methyltransferase, followed by incubation with alkaline phosphatase). Lower panel: enlarged view of the chromatogram from 25.9- 29 min. IS: Internal standard; #4, #5, and #6 designate new and yet unidentified compounds from L239A and C241A mutant enzymes. All non-labeled peaks were also present in the control assay performed with the corresponding denatured enzyme. Number of replicates: n = 3-5.


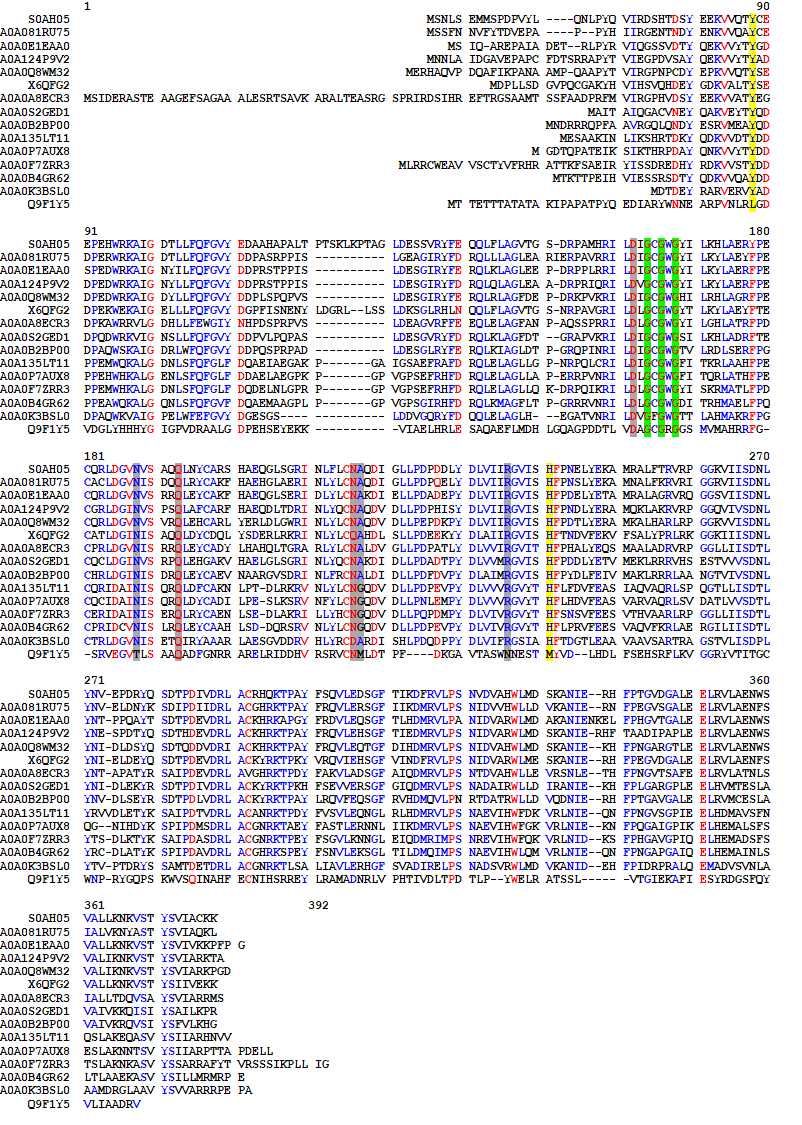


**Figure S14: Sequence alignment (BLOSUM62) of 14 annotated microbial methyltransferases.** Proteins derived from different species of bacteria that showed the highest sequence identity (Uniprot BLAST search ^79^) to the *Serratia plymuthica* (SOD_c20760) FPPMT were selected. The multiple sequence alignment was performed using Multalin ^80^ in order to determine highly conserved regions. GPPMT from *Streptomyces coelicolor* was added to the alignement due to its similar substrate to the FPPMT, although the sequence identity to the other proteins was lower ^36^. Highly conserved residues are colored in red, less conserved residues in blue. The putative catalytic dyad of Y39 and H191 is highlighted in yellow, the SAM binding motif GxGxG ^37^ in green, and further amino acids interacting with the co-factor in light grey ^81,82^. Uniprot IDs and organisms: S0AH05 *Serratia plymuthica* (*SOD_c20760* (FPPMT)); A0A081RU75 *Photorhabdus temperata;* A0A0E1EAA0 *Pseudomonas chlororaphis*; A0A124P9V2 *Burkholderia sp.*; A0A0Q8WM32 *Achromobacter sp.*; X6QFG2 *Providencia alcalifaciens*; A0A0A8ECR3 *Streptomyces sp.*; A0A0S2GED1 *Lysobacter gummosus;* A0A0B2BP00 *Mumia flava;* A0A135LT11 *Penicillium patulum*; A0A0P7AUX8 Neonectria ditissima; A0A0F7ZRR3 Hirsutella minnesotensis; A0A0B4GR62 *Metarhizium guizhouense;* A0A0K3BSL0 *Kibdelosporangium sp.*; Q9F1Y5 *Streptomyces coelicolor.*


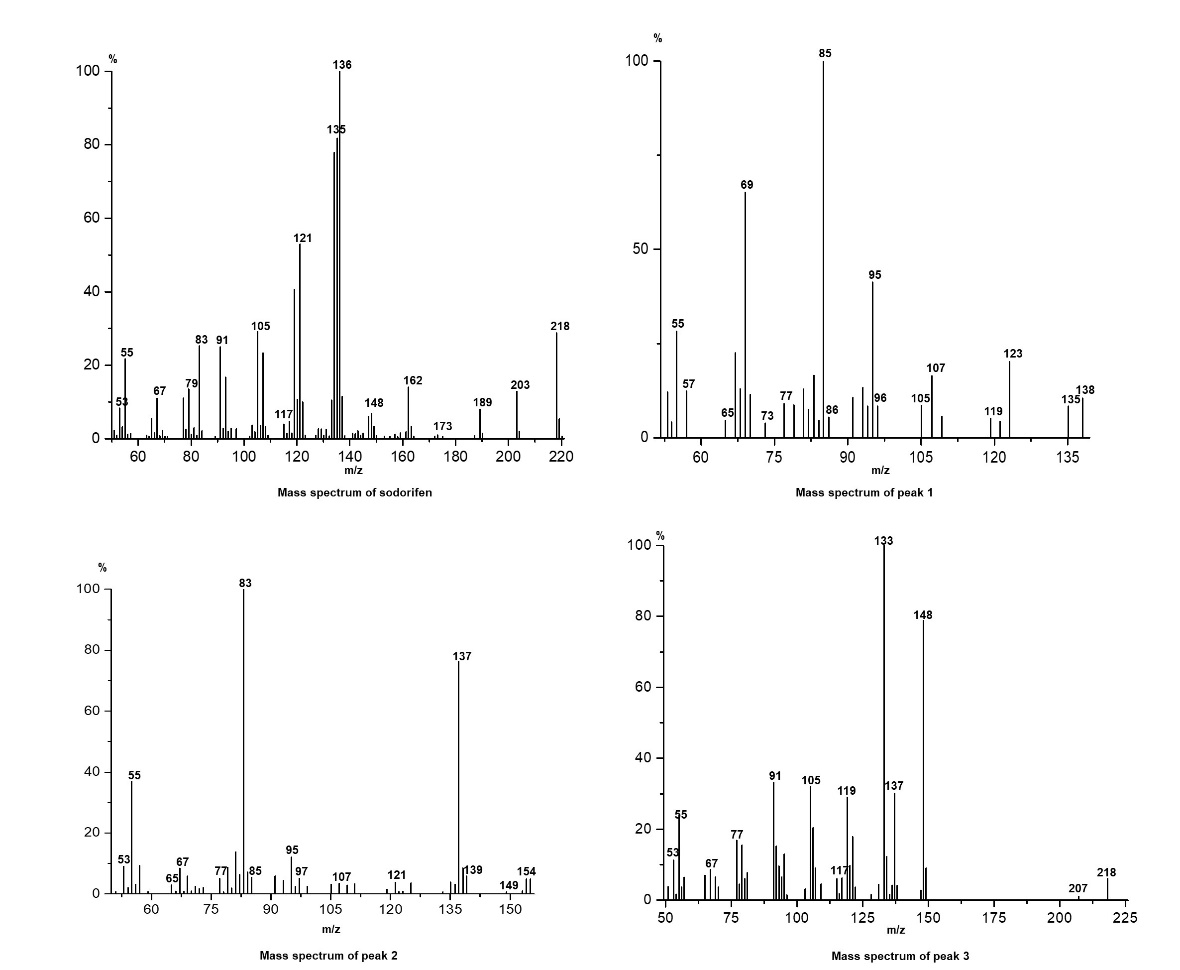


**Figure S15: Mass spectra of compound #1, #2 and #3.** The compounds (Fig. S11) were present in the TIC when the *Serratia plymutica* FPP *C*-methyltransferase L239A and C241A mutant enzymes were analyzed in double enzyme assays (FPP incubated with the mutant or wildtype FPP methyltransferase and sodorifen terpene synthase (SODS)). The mass spectrum of sodorifen after double enzyme assay is shown (RI = 1379) and the retention indices (RI) of the new compounds are RI = 1640 (**#1**)**,** RI = 1662 (**#2**) and RI = 1577 (**#3**).


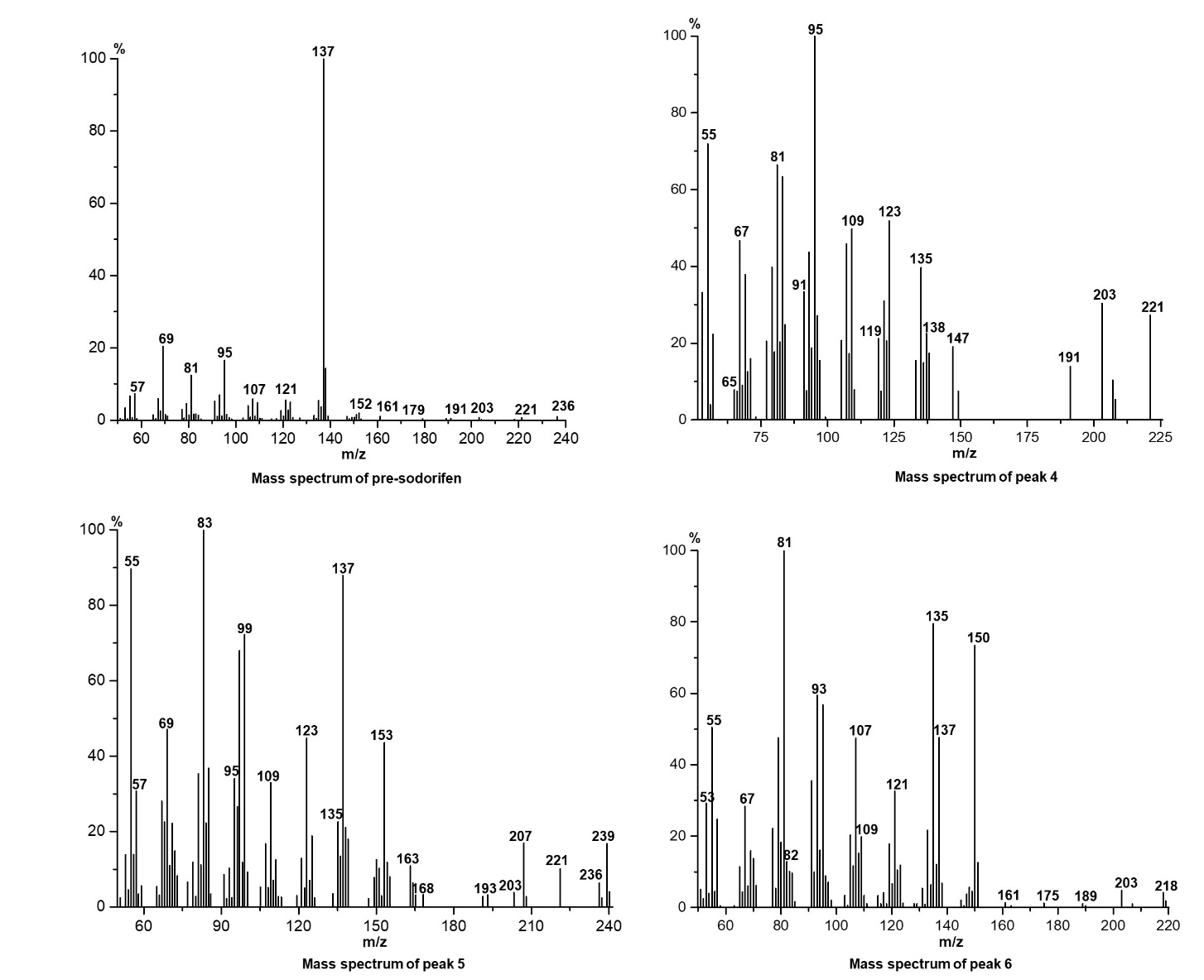


**Figure S16: Mass spectra of compound #4, #5 and #6.** The compounds (Fig. S12) were present in the TIC when the *Serratia plymutica* FPP *C*-methyltransferase L239A and C241A mutant enzymes were analyzed in coupled enzyme assays (FPP incubated with the mutant or wildtype FPP methyltransferase, followed by incubation with alkaline phosphatase). The mass spectrum of pre-sodorifen after coupled assay is shown (RI = 1722) and the retention indices (RI) of the new compounds are RI = 1827 (**#4**)**,** RI = 1949 (**#5**) and RI = 1853 (**#6**).


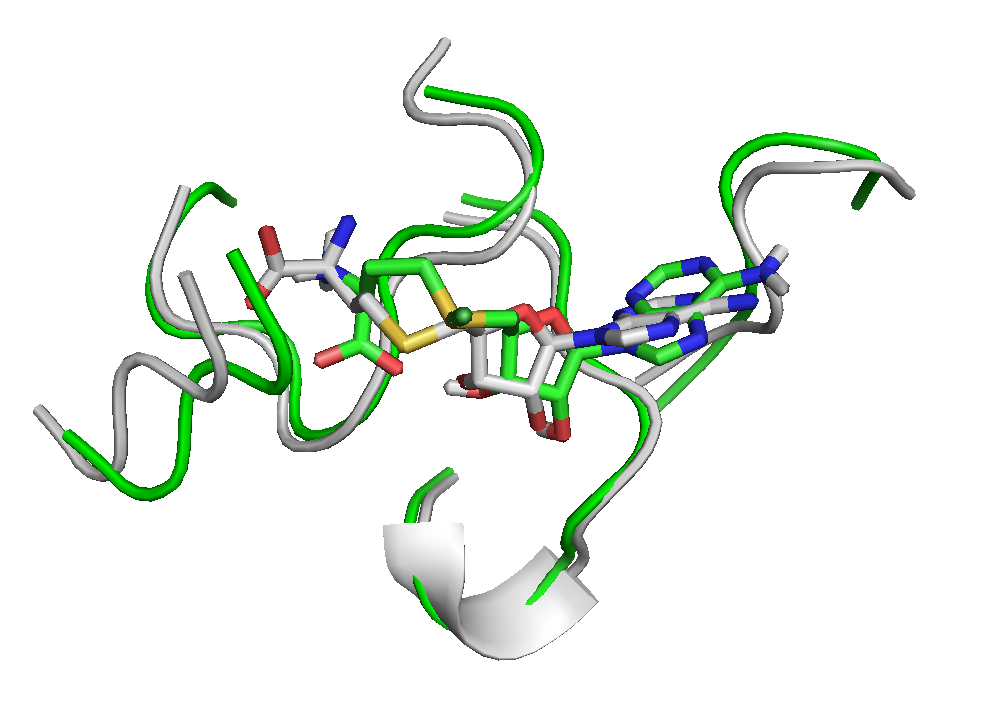


**Figure S17: Superposition of the SAH binding site of 5KOK (grey backbone secondary structure) with the model of the FPP *Serratia plymuthica* FPP *C*-methyltransferase (green).**


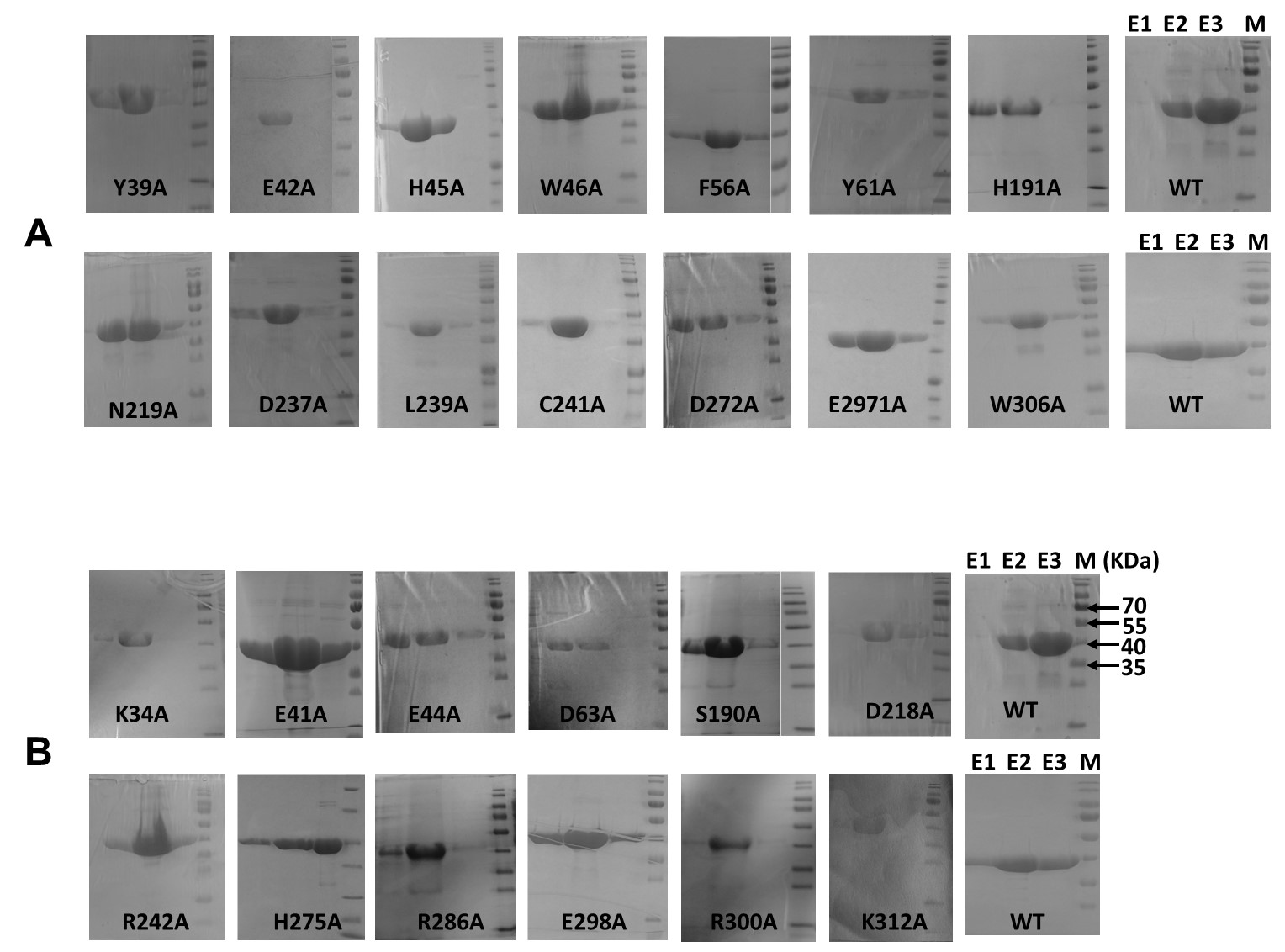


**Figure S18: Documentation of the SDS-PAGE gels of IPTG induced samples of *E. coli* BL21(DE3) cultures of the wild type and all mutant of *Serratia plymuthica* FPP** C**-methyltransferases.** After cell lysis, the overexpressed protein in the crude extract was purified by Ni-NTA affinity chromatography. **(E1)** Elution fraction1, **(E2)** elution fraction2, **(E3)** elution fraction3, **(M**) marker. The purified proteins were stored at -20 °C or -70 °C for further use. **A)** SDS-PAGE gels of all sodorifen or pre-sodorifen non producing mutants. **B)** SDS-PAGE gels of all sodorifen or pre-sodorifen producing mutants.
